# PhysioSpace: Relating Gene Expression Experiments from Heterogeneous Sources Using Shared Physiological Processes 

**DOI:** 10.1371/journal.pone.0077627

**Published:** 2013-10-17

**Authors:** Michael Lenz, Bernhard M. Schuldt, Franz-Josef Müller, Andreas Schuppert

**Affiliations:** 1 Aachen Institute for Advanced Study in Computational Engineering Science, RWTH Aachen University, Aachen, Germany; 2 Zentrum für Integrative Psychiatrie, Kiel, Germany; 3 Bayer Technology Services GmbH, Leverkusen, Germany; National Institute of Genomic Medicine, Mexico

## Abstract

Relating expression signatures from different sources such as cell lines, in vitro cultures from primary cells and biopsy material is an important task in drug development and translational medicine as well as for tracking of cell fate and disease progression. Especially the comparison of large scale gene expression changes to tissue or cell type specific signatures is of high interest for the tracking of cell fate in (trans-) differentiation experiments and for cancer research, which increasingly focuses on shared processes and the involvement of the microenvironment. These signature relation approaches require robust statistical methods to account for the high biological heterogeneity in clinical data and must cope with small sample sizes in lab experiments and common patterns of co-expression in ubiquitous cellular processes. We describe a novel method, called PhysioSpace, to position dynamics of time series data derived from cellular differentiation and disease progression in a genome-wide expression space. The PhysioSpace is defined by a compendium of publicly available gene expression signatures representing a large set of biological phenotypes. The mapping of gene expression changes onto the PhysioSpace leads to a robust ranking of physiologically relevant signatures, as rigorously evaluated via sample-label permutations. A spherical transformation of the data improves the performance, leading to stable results even in case of small sample sizes. Using PhysioSpace with clinical cancer datasets reveals that such data exhibits large heterogeneity in the number of significant signature associations. This behavior was closely associated with the classification endpoint and cancer type under consideration, indicating shared biological functionalities in disease associated processes. Even though the time series data of cell line differentiation exhibited responses in larger clusters covering several biologically related patterns, top scoring patterns were highly consistent with a priory known biological information and separated from the rest of response patterns.

## Introduction

In many biological and medical research fields, such as stem cell research, drug development or analysis of disease status, it is important to integrate data from different sources, such as cell lines, in vitro cultures from primary cells or clinical biopsies. Data integration has the possibility to combine the knowledge derived from different experiments, providing a bigger picture surrounding the new data and improving the interpretation of results [1]. However, biological heterogeneity in clinical samples, lab dependent effects as well as technical noise challenge the direct integration of data from heterogeneous sources. Furthermore, the typical low number of replicates in lab experiments, especially for time series analyses, complicates the statistical significance analysis. 

Data integration approaches have been implemented on different levels using gene expression data. The classical analyses started with the integration on a single gene level, e.g. by interpreting differential gene expression in newly performed experiments using knowledge from gene annotation databases. These analyses were then extended to sets of genes, corresponding to specific biological functionalities, pathways or genomic locations [2-4]. The gene set analysis summarizes the information of several genes, providing a broader view on the gene expression changes with better interpretability in terms of intracellular pathways and functionalities. A further step into this direction is a whole genome based comparison of phenotypical changes, linking the gene expression changes in the newly performed experiments to gene expression patterns that are associated with specific tissues, clinical parameters, or changes in the cellular environment [5-7].

This last step has been implemented by extension of gene set enrichment analyses to include signatures derived from high-throughput experiments [3], explicitly focusing on oncogenic or immunologic phenotypes as well as by signature association approaches relating experiments in drug response databases [8] with the goal to identify biologically meaningful connections between observed phenotypes [5,9]. 

The present article, in contrast, focuses on the relation of gene expression changes to various tissue or cell type specific expression patterns. This specific focus becomes increasingly relevant as outlined by the following two examples. First, differentiation of pluripotent stem cells towards neural cells or cardiomyocytes, for instance, is anticipated to bear enormous potential for drug screening and regenerative medicine [10]. In order to properly characterize these in vitro differentiated cells and their differentiation dynamics, it is essential to compare them to the respective primary tissue on a whole genome gene expression level [6,7,11]. Second, different disease stages in cancer have been linked to stem cell signatures [12-14], suggesting an earlier developmental state of cancer cells compared to normal cells [15]. However, it is important to evaluate such signature associations properly in order to avoid misinterpretations. Counterintuitively, microarray analysis of Breast cancer data have revealed [16,17] that diverse gene sets and signatures do possess largely the same predictive quality and that variation in the predictive performance can be explained by variation in the contribution of a single passenger proliferation signature. Moreover, the focus of cancer research has shifted from a narrow focus on the genetic and molecular properties of the primary cancer cell line to the integration of the surrounding tumor environment [18].

Global analyses of gene expression patterns across diverse tissues and cell lines are typically performed in an unsupervised way, e.g. based on principal components analysis (PCA) [19,20]. PCA identifies orthogonal directions of highest variance in the data. While principal components are often associated with known phenotypes, uncovering these associations requires additional efforts since this is not explicitly built into the algorithm.

The presented PhysioSpace method serves as an exploratory research tool that allows getting a large scale overview of the data in terms of defined physiological coordinates. PhysioSpace complements single gene based analyses, gene set and pathway methods and unsupervised global methods like PCA.

The PhysioSpace algorithm defines directions (signatures) in a supervised way based on retrospective microarray data. These directions are directly associated with specific phenotypes defined by data postprocessing.

**Figure 1:**
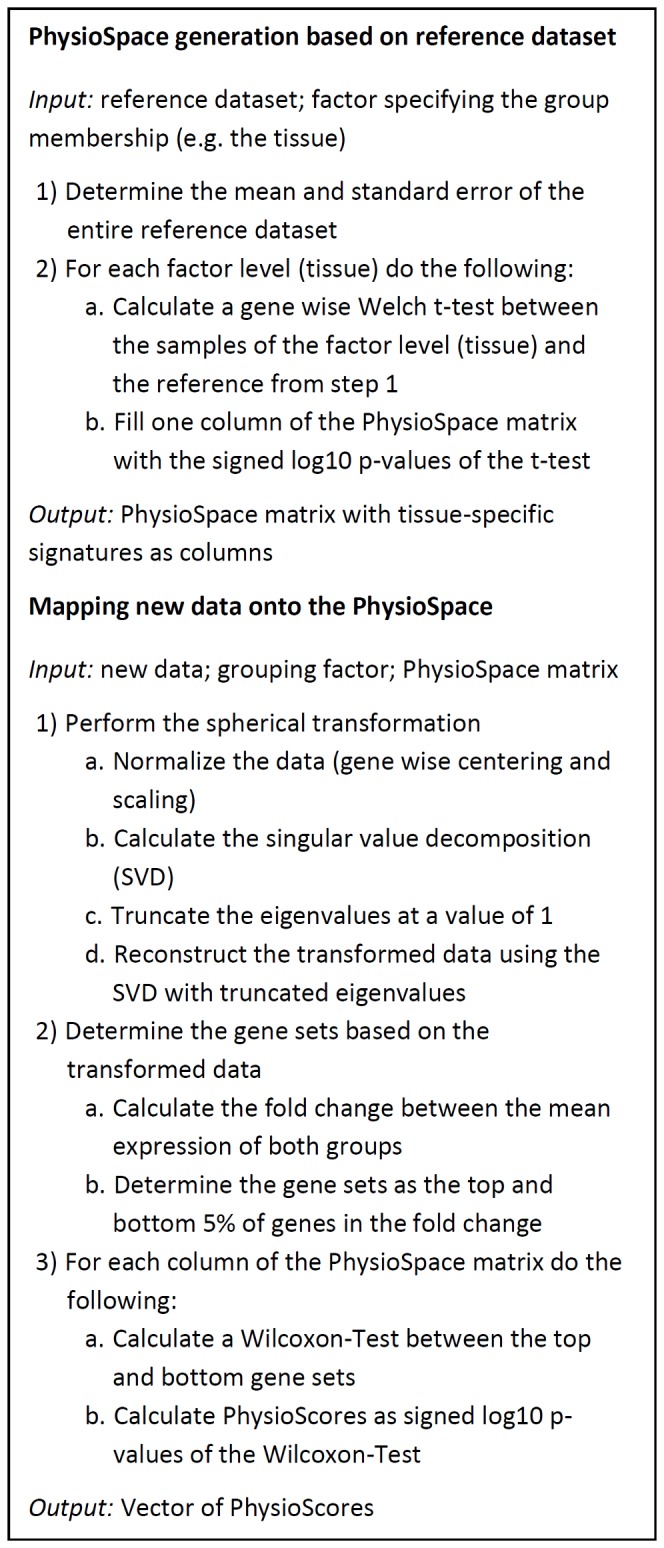
Main steps of the PhysioSpace generation and mapping algorithms.

The directions are derived by comparing samples of a specific tissue with a reference via a t-test (Figure 1, Materials and Methods). In this article, the reference is computationally determined as the mean over a large set of samples from various tissues and cell lines. This is similar to centering in principal components analysis and makes it easier to compare the diverse set of conditions discussed later. Experiment specific references can be used for more narrow applications.

The differential expression between samples from a specific tissue and this reference is then used as a signature representing the characteristic expression pattern of this tissue. Due to the apparent similarity of different tissues, e.g. neural tissues from different regions of the brain or different tissues related to the immune system, some PhysioSpace signatures are highly correlated (Figure S1 in File S1). Besides these obvious correlations between tissues with similar functions, there are also some dependencies of tissues or cell lines due to more general shared processes like proliferation. The large amount of correlations between diverse tissues (Figure S1 in File S1) reflects the low dimensionality of large scale gene expression as observed in principal components analysis of a huge dataset [19] and in the ability to reprogram cells from a somatic to a pluripotent state with only a few reprogramming factors [21]. Furthermore, tissues are usually mixtures of different cell types and some cell types, fibroblasts for instance, are present in many different tissues.

**Figure 2:**
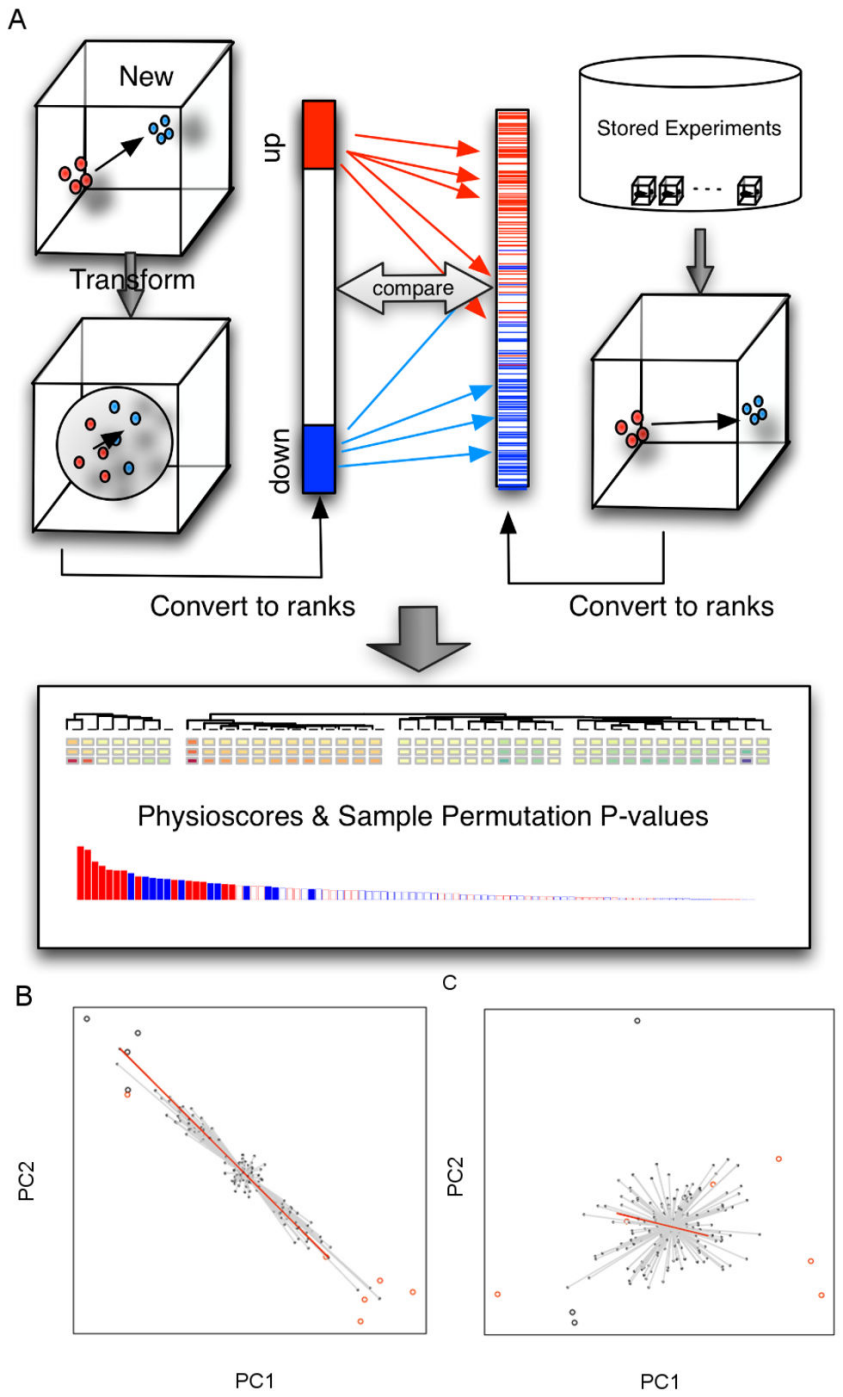
Overview over the PhysioSpace algorithm. (A) Data from a new experiment is transformed to remove ellipticity and the resulting fold-change vector is compared to a compendium of signatures from prior experiments using a robust, rank-based scoring method. Graphical displays and the statistical validation allow to evaluate the position of the new experiment in the global PhysioSpace. (B, C) Illustration of the influence of non-sphericity on sample permutations. (B) In the presence of a strong ellipticity, sample permutation does not randomize directions in contrast to more spherically distributed samples as obtained through the spherical transformation approach (C).

The task of mapping gene expression changes into the PhysioSpace can be defined in the light of high dimensional gene expression spaces as follows: A phenotype is interpreted as a point or cloud in an expression space and phenotypical change is a vector connecting the centers of different clouds in the same expression space (Figure 2 A). In microarray experiments this vector can in most cases be identified with the vector of fold-changes. Mapping of a differential expression vector in an experiment to the PhyisoSpace is a comparison of the vector of fold changes in the experiment to differential expression vectors associated with known phenotypes spanning the PhysioSpace. In this interpretation, the PhysioSpace method reduces to the robust identification of vectors that point in similar directions within the gene expression space.

Considering that the PhysioSpace method should be able to compare data from heterogenous sources, derived from cell lines, in vitro cultures from primary cells, or primary patient biopsies, it is important to use robust and statistically sound techniques. In this article we follow practices from gene set analysis [3,22,23], namely, focusing on informative genes, and using rank based statistical methods in order to achieve robustness. A spherical transformation of the data [24] is applied as preprocessing step in order to reduce the non-phenotype specific variation and to facilitate proper statistical assessment via sample label permutation. Two gene sets of up- and down-regulated genes are defined and used for enrichment analysis of the PhysioSpace signatures via the Wilcoxon rank-sum test [22] (Figure 1, Figure 2 A, Materials and Methods). The signed log_10_ p-values from the Wilcoxon test, termed PhysioScores, are then used for visualization purposes. If there are at least 9 samples in each group, sample-label permutation is performed to assess the significance of the PhysioScores. 

The algorithm used in this article is similar to classical gene set enrichment algorithms. However, the usage of signatures instead of gene sets allows to perform the enrichment calculation in a backward direction, defining the gene sets on the new data and calculating the enrichment on the tissue specific signatures. This backward direction provides a different view on the data as evaluated and discussed below. A similar backward approach has been used previously [5,25]. Compared to these implementations, the main innovation of our algorithm is the usage of a spherical transformation, improving the results especially in the case of high heterogeneity in the data.

We evaluate the performance of the PhysioSpace method and discuss the effect of the spherical transformation by analysis of simulated mixtures of embryonic stem cells (ESCs) with different tissues. We then apply the method to analyze tumor development comparing different breast cancer grades and prostate Gleason scores, as well as to investigate the effect of smoking on gene expression of lung cancer tissues. These examples show three principally different outcomes of the PhysioSpace method that are used to exemplify possible interpretations. Furthermore, the cancer data are utilized to investigate the relationship between PhysioScores and permutation p-values, providing useful information for the applicability of PhysioSpace in the case of low numbers of replicates. The PhysioSpace method is then applied on tracking of induced pluripotent stem cell (iPSC) differentiation experiments towards neural cells, cardiomyocytes and trophoblast lineages in a physiological context. It detects the direction and dynamics of differentiation, uncovering interesting information from data with very small numbers of replicates, and matches well to biological expectations. The comparison to a classical forward enrichment algorithm is performed on the cancer, differentiation, and simulated data, with overall slightly better results for the implemented algorithm. The robustness of the proposed method is demonstrated by the use of different PhysioSpaces (table 1) and datasets across microarray platforms. 

**Table 1:** Datasets for the PhysioSpace generation.

Accession	Usage	Description
GSE7307	PhysioSpace 1	677 samples corresponding to 93 different tissues or cell lines
GSE23402	PhysioSpace 1	17 ESC samples (the 25 hiPSC and Fibroblast samples are not used)
GSE2361	PhysioSpace 2	36 samples, each from a different tissue
E-MTAB-62	PhysioSpace 3	5372 samples divided into 369 different groups as annotated in [18]

## Results

### A spherical transformation improves the PhysioSpace performance

Clinical datasets often suffer from large, non-phenotype associated variation, affecting the determination of fold change vectors. This can lead to spurious associations, especially in the case of relatively small sample sizes and large heterogeneities in the data. The spherical transformation (Figure 1, Materials and Methods) results in a homogenization of the data, reducing the effect of very large non-phenotype associated variations. Thus, it leads to increased sensitivity and specificity in the case of high non-phenotype associated variation and comparably low signal strength. 

In the opposite case, i.e. when the heterogeneity in the data is considerably lower than the effect of interest, the sample permutation approach does not generate a meaningful null distribution. Random re-sampling of sample labels generates new vectors connecting the centroids of the two sampled groups. In the case of elliptical data distributions the sampled vectors are highly correlated, resulting in a decreased significance (Figure 2 B). The spherical transformation diminishes this effect, resulting in a null distribution of vectors resembling an approximately spherical distribution within the lower dimensional space spanned by the data (Figure 2 C). 

In order to investigate the two described effects of the spherical transformation, two datasets of embryonic stem cells (GSE33789) and cancerous and normal lung tissues (GSE19804) were downloaded, normalized and merged (table 2). Dataset GSE33789 contains 10 embryonic stem cell (ESC) samples, which were used as a homogenous dataset, i.e. with relatively low amounts of variability and confounding effects. Dataset GSE19804 is more heterogeneous, consisting of 60 lung cancer samples and 60 samples of adjacent normal lung tissue.

**Table 2:** Datasets used for simulations.

Accession	Usage	Description
GSE33789	All simulations	10 ESC samples (2 Fibroblast samples are not used)
GSE19804	Effect of spherical transformation	60 lung cancer and 60 adjacent normal lung samples
GSE18676	Mixture simulations	24 samples from 22 different tissues and 2 cell lines

**Figure 3:**
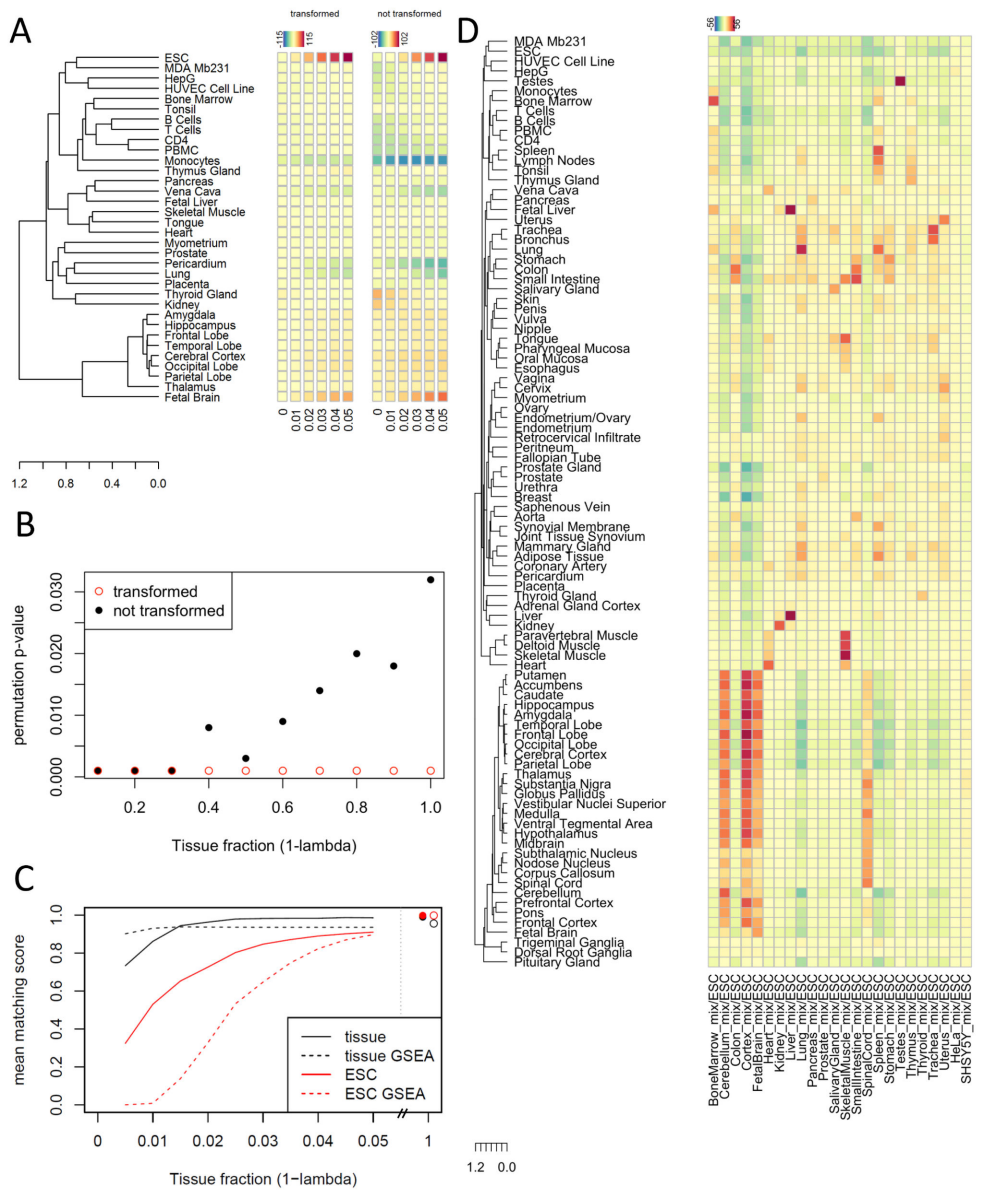
Performance evaluations with simulated data. (A) In the case of relatively high heterogeneity and comparably low signal strength, the spherical transformation increases sensitivity and specificity of the simulated effect, i.e. results in a strong and specific increase of the ESC score (left part). The results without spherical transformation (right part) are more heterogeneous. (B) The null-distribution obtained from re-sampling without spherical transformation is not meaningful in cases of high signal strength and low heterogeneity, leading to increasing p-values for increasing signal strength in simulated data. This effect does not occur when the spherical transformation is applied. Depicted are permutation p-values for the lung signature with (red dots) and without (black dots) spherical transformation. (C) The mean matching score of 21 simulated mixtures is compared between the implemented PhysioSpace algorithm and a classical GSEA based method. The matching score is defined as the quotient of the respective tissue (or ESC) score and the highest (lowest) score. It is truncated at a minimum of zero, avoiding negative values. While there are some differences between the two methods, especially for very low mixture values, the overall performance does not generally favor one or the other algorithm. (D) The PhysioScores of simulated mixtures of 97% ESCs with 3% of different tissues are visualized as an exemplary case, showing a very nice agreement with biological expectations. Correlations between different signatures are represented by the dendrogram on the left hand side as well as by simultaneous increasing PhysioScores in the columns of the heatmap-like representation.

Two different types of simulated data were produced for investigation of the above described effects. First, 40 samples were randomly drawn from the lung dataset GSE19804. No distinction of normal and cancerous lung tissue was made. Cancerogenicity was rather interpreted as an unknown confounding effect, increasing the heterogeneity of the dataset. The first 20 of these samples were subjected to a computational modification, simulating a mixing of lung tissue with ESCs with mixing factor *λ*=0,0.01,…,0.05 (Materials and Methods). For each value of*λ*, the mixed samples were then compared to the remaining 20 lung samples via the PhysioSpace method with and without spherical transformation. The analysis results with spherical transformation show less confounding effects, i.e. a more specific increase of the ESC score (Figure 3A, Figure S2 in File S1). Furthermore, the spherical transformation increases the sensitivity, having already a relatively high ESC score for a mixing fraction of 2% ESCs (PhysioScore of 41.3), while the ESC score without transformation has a value of 19.3 for *λ*=0.02 and 48.7 for*λ*=0.03. The permutation p-values confirm the higher sensitivity of the analysis with spherical transformation showing significant test results for the ESC score with 4% and 5% ESC in the mixtures, while the analyses without transformation show no significant result.

In the second simulation, the low-heterogeneity ESC dataset was analyzed, where 10 samples were simulated as mixtures of ESC and adjacent normal lung tissue (Materials and Methods) and compared to unmodified ESC samples. In order to simulate a rather strong signal, the fraction of lung tissues in the mixture was set to 0.1, 0.2,…, 1. For the analysis without spherical transformation, the negative effect of increasing ellipticity in the data can be observed from Figure 3B, showing a non-meaningful increase of the p-value associated with the lung signature for increasing fractions of lung tissue in the mixtures. In contrast, for the analysis with spherical transformation, the corresponding p-value stays at the lowest possible value that can be achieved with 1000 permutations (Figure 3B).

### Evaluating PhysioSpace performance using simulated mixtures

In order to evaluate the ability of the PhysioSpace method to detect changes in tissue composition, the analysis of simulated mixtures was extended to several different tissues. For this purpose, the dataset GSE18676 was considered, consisting of 22 different tissues and 2 cell lines. Each tissue or cell line is represented by a single sample only. The 24 samples were computationally mixed with one ESC sample from dataset GSE33789 with a mixture proportion of 97% ESC and 3% of the tissue sample and compared to the remaining 9 ESC samples (Figure 3 D). This analysis again reveals the correlations that are present between PhysioSpace signatures, most prominently between signatures corresponding to the diverse brain regions. Furthermore, the absolute values differ between the 22 tissue samples, being relatively low for Pancreas or Prostate, for instance, and highest for Testes. However, the ranking of PhysioSpace signatures is highly concordant with the known biological information. In 18 out of 22 cases (excluding the 2 cell lines), the top-ranking signature exactly matches the tissue used to simulate the mixture. The 4 exceptions are Cortex, Fetal Brain, Pancreas, and Spinal Cord. For Cortex, there is no PhysioSpace signature with an exact matching name, but the top ranked scores are biologically plausible. The highest PhysioScore corresponds to Frontal Lobe followed by further signatures associated with specific regions of the brain. For Pancreas, the top-ranked signature corresponds to Small intestine (PhysioScore of 14.49), directly followed by Pancreas (13.78) and separated from the other scores starting with Colon (5.76). The top-ranked scores for the Spinal Cord mixture, i.e. Medulla (28.99), Spinal Cord (28.87), and Substantia Nigra (28.19), are also biologically plausible. Only the fetal brain mixture partially leads to unexpected results. The top-ranked scores correspond to adult brain regions and the fetal brain signature has rank 6 only. A possible reason for this result may be different gestational ages of the fetal brain sample used to develop the signature and the fetal brain sample used for the mixture analysis. It has been reported [26] that the gene expression of neural tissues changes strongly during fetal development and global gene expression patterns of late fetal brain lie between those of neural tissue from earlier gestational ages and from postnatal brain. Detailed information about the gestational ages of the fetal brain samples is not available.

Overall, these results reflect the simulated changes very well, indicating high robustness of the PhysioSpace method even in cases where data from different studies were combined as well as high sensitivity and specificity for detecting mixtures with mixture fractions of only 3%. Following an advice of a referee, we set out to test the dependence of the matching on the mixing fraction lambda and compared the results to those using a typical gene set enrichment algorithm as implemented in the geneSetTest method of the limma package [27] in R. The geneSetTest algorithm takes a gene set and a differential expression vector as input and performs a competitive enrichment test, evaluating whether the genes in the specified set are more differentially expressed than randomly selected genes. Here, we use the 5% highest ranked genes in the PhysioSpace signatures as gene set and the fold change as measure of differential expression. This is an example of a classical forward GSEA approach in contrast to the presented backward approach.

For the performance assessment, two matching scores were calculated, a tissue matching score and an ESC matching score. They are defined as the ratio of the expected PhysioScore, i.e. of the matching tissue (or the ESCs), and the highest (or lowest) PhysioScore. For example, for the mixing of the bone marrow sample with the ESC sample, the tissue matching score is the ratio between the bone marrow PhysioScore and the highest PhysioScore. This matching score is 1 in case of a perfect match, e.g. if the bone marrow score has the highest value itself, and gradually decreases with the distance of the expected score to the actual highest (lowest) score. We truncated the score at zero to avoid negative values. In Figure 3 C, the mean matching score of the 21 tissues, having a corresponding PhysioSpace signature, is plotted over 1-lambda in the range from 0.005 to 0.05 in steps of 0.005. The tissue matching score is nearly constant for a mixing fraction larger than 0.015, while the ESC matching score is increasing for a relatively wide range of lambda. The performance of the two alternative mapping algorithms (GSEA and our method) is comparable, with an advantage of GSEA in the tissue matching score for low mixture fractions and some advantages for our method in the other cases (Figure 3 C).

### Application to cancer data

Cancer is a highly heterogeneous disease consisting of different subtypes traditionally defined by specific histological markers like grade in breast cancer or Gleason score in prostate cancer [18]. In recent years, attempts to define different cancer subtypes based on gene expression patterns were realized in order to improve medical treatment in the framework of personalized medicine or to refine prediction of therapy outcome. Due to some functional similarities of cancer cells to normal stem cells, stem cell signatures have been proposed for several cancer types, including breast and prostate cancer, to define subtypes with different prognosis [12-14,28]. However, for the case of breast cancer, it has been argued that most random signatures are significantly associated with outcome [16], dominated by a proliferation signature involving a large number of genes. Therefore, it is important to complement statistical evaluation of prediction accuracy by methods comparing diverse biologically realistic patterns in a global perspective before drawing conclusions on possible mechanisms or therapeutic directions.

**Figure 4:**
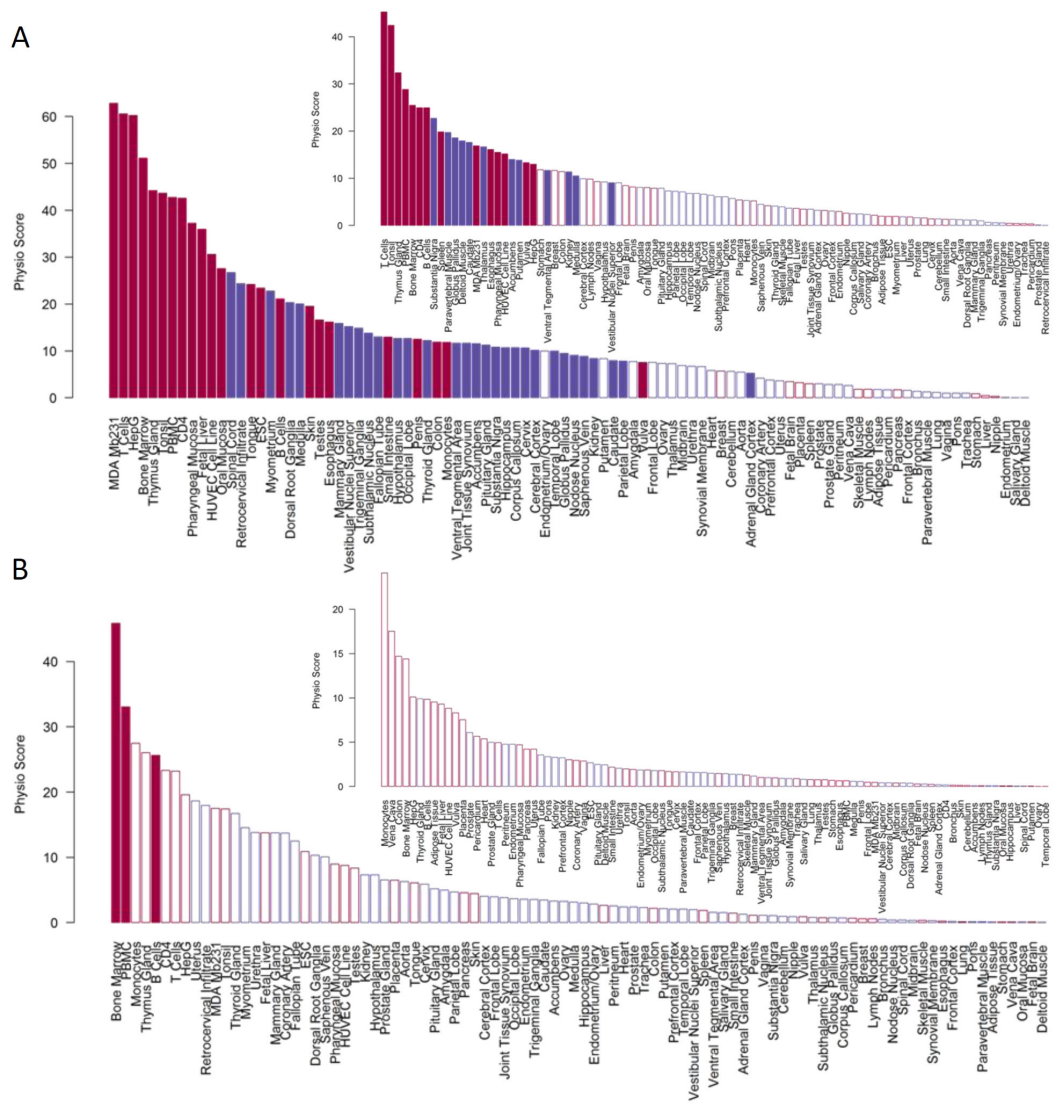
Details of results from cancer progression. Ranking of PhysioScores comparing breast cancer samples of grade 1 to grade 2 or 3 (A) and lung samples from never smokers to former or current smokers (B). Apparently, grading differences in breast cancer are associated with more signatures from the PhysioSpace than differences in gene expression of smokers and non-smokers. Blue (red) colors depict negative (positive) PhysioScores. Filled bars indicate significant scores according to a sample-permutation FDR (Benjamini-Hochberg) cutoff of 0.1.

In this context, the PhysioSpace method was applied to investigate differences in global gene expression between breast cancer grades. A dataset with 189 breast cancer samples (GSE2990) was analyzed, incorporating 64 breast cancers of grade 1, 48 of grade 2, 55 of grade 3, and 22 with missing information on breast cancer grade. The vectors of differential expression of grade 1 to that of grade 2 and grade 3 cancers were compared to the signatures in the PhysioSpace, resulting in a large number of significantly associated reference signatures (Figure 4 A). The dominating PhysioScores correspond to signatures of immune and bone marrow derived cells as well as to the breast cancer cell line MDA-Mb231 and the hepatic cancer derived cell line HepG. Further significant scores include embryonic stem cell (ESC), HUVEC cell line, and fetal liver signatures. The large number of significant scores (see also Figures S5, S6, and S7 in File S1) corresponding to rather different phenotypes suggests that the gene expression shift does not only represent cell type specific processes. There seems to be a common underlying mechanism that is shared by most of the significant phenotypes, most probably corresponding to proliferation [16,29]. 

**Figure 5:**
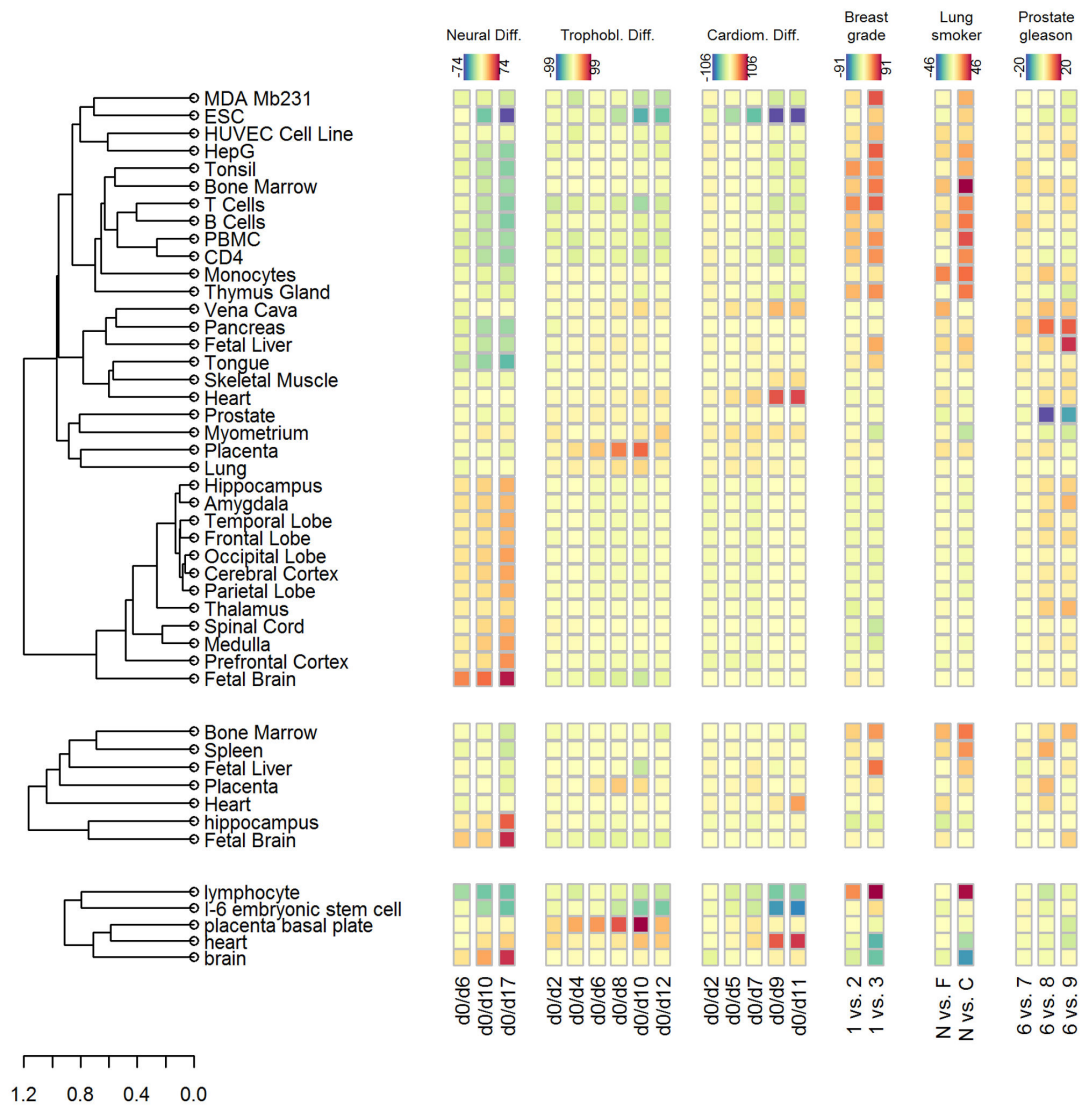
Heatmap of PhysioScores combining selected signatures from three PhysioSpaces. The PhysioScores for all six investigated datasets are visualized in context for selected signatures. In order to evaluate the stability of the method, the signatures were derived from three different physiological databases resulting in PhysioSpaces 1-3. The results are depicted in a heatmap-like representation. The color scheme differs between datasets but is the same for the PhysioSpaces 1-3, ranging from negative values in blue and green to positive values in orange and red. The dendrogram represents a hierarchical clustering of the signatures according to a Pearson-correlation distance. Values within clusters are usually similar, e.g clusters of neural or immune signatures. The results corresponding to PhysioSpaces 1-3 show similar dynamics and consistent dominating signatures, while the absolute values are only approximately comparable.

In contrast to the breast cancer results, the influence of smoking on gene expression seems to be more phenotype specific. A comparison of the gene expression of (normal and cancerous) lung tissues from 31 never to 36 former, and 40 current smokers (dataset GSE10072, table 3) revealed a significant increase in immune signatures for current smokers (Figure 4 B, Figure 5). This is in accordance with the known increase of immune cells, especially macrophages, in the lung caused by cigarette smoke [30] and with previous gene expression analyses of lung adenocarcinomas [31]. The results shown in the present article were obtained using cancerous and adjacent normal lung tissues together in a single analysis. The increased heterogeneity in the data due to the joint use of cancerous and normal tissues is, as in the simulation example above, not critical for the performance of the PhysioSpace method. The results using only cancerous or only normal tissues are fairly similar (data not shown), showing the robustness of our method due to the spherical transformation. 

**Table 3:** Cancer datasets.

Accession	Usage	Description
GSE2990	Breast grade	Breast cancer samples of grade 1 (64), 2 (48), or 3 (55); the 22 samples with missing information on breast cancer grade are not used
GSE10072	Lung smoker	107 samples from cancerous or adjacent normal lung tissue from never (31), former (36), or current (40) smokers
GSE21034	Prostate Gleason	131 primary prostate cancer samples with a Gleason score of 5 (1) 6 (77), 7 (42), 8 (7), or 9 (4); 19 metastases; normal prostate samples and cell lines are not used
GSE16560	Prostate Gleason	281 primary prostate cancer samples with a Gleason score of 6 (83), 7 (117), 8 (27), 9 (49), or 10 (5).

The third analyzed dataset consists of prostate tumors with differing Gleason scores. Dataset GSE21034 consists of primary prostate tumors, metastases, and prostate cancer cell lines. In order to concentrate on the differences associated with Gleason score, only primary tumor samples were considered for the analysis. The highest scoring PhysioScores are fetal liver and pancreas, increasing with Gleason score, as well as prostate, decreasing with Gleason score (Figure 5). Statistical validation via sample label permutation, however, reveals that there are likely no detectable significant signatures present in the dataset analyzed by us, with the lowest adjusted p-value (Benjamini-Hochberg [32]) being 0.57. This example shows the importance of rigorous statistical validation in order to avoid false positive results as frequently obtained through gene label permutation [33]. A first hint on the non-significant results may have been obtained by the observable lower PhysioScores, compared to the previous analyses (Figure 5). However, it is generally not possible to define a rigorous significance threshold for the PhysioScores, since their absolute value depends on the size and correlation structure of the gene sets [33].

We applied the PhysioSpace method additionally to compare metastases and primary tumors in dataset GSE21034. The result of this analysis is dominated by a strongly negative prostate-signature (Figure S3 in File S1). This is reasonable, since metastases are not located in or close to the prostate, in contrast to primary prostate tumors. The positive associations are dominated by immune and cell line scores including a rather weak, but significant association with the ESC-signature (PhysioScore of 12.6, adjusted p-value of 0.024). 

Furthermore, we analyzed dataset GSE16560, in order to investigate whether differences in prostate cancer samples of different Gleason scores have any physiological interpretation. In this analysis, a weak association between Gleason score and cell line signatures, including the ESC signature, can be found (Figure S3 in File S1). The corresponding adjusted permutation p-values for the ESC signature show a trend towards higher Gleason scores (p = 0.22, 0.11, 0.32, and 0.21 for Gleason scores 6 vs 7, 8, 9, and 10, respectively). 

Markert et al. [12] describe an association between Gleason score and an ESC signature for datasets GSE16560 and GSE21034. We were not able to confirm this association with our method. One explanation for the apparent disagreement may be the fact that Markert et al. used not only primary tumors, but also metastases for their analysis of dataset GSE21034 [12]. We also found a significant association of our ESC signature with the differential expression between metastatic and primary tumors. Another explanation might be that Markert et al. use a rather small gene set. PhysioSpace and gene sets therefore may provide complementary insights into the same experimental data.

In summary, the three presented application examples show three qualitatively differing outcomes of the PhysioSpace method. Breast cancer grade is significantly associated with many signatures corresponding to various cellular phenotypes, suggesting a common underlying mechanism. The effect of smoking on lung cancer is more specific, showing primarily an increase in immune signatures. Finally, Gleason scores in prostate cancer show no significant associations for dataset GSE21034.

In order to investigate the effect of the spherical transformation with real data, the permutation p-values were compared to the PhysioScores for all cancer data, with and without spherical transformation (Figure S4 in File S1). From this analysis it is evident that the spherical transformation leads to an almost linear relation between the PhysioScore and the logarithmic permutation p-values. Without spherical transformation, the two scores are less comparable, with strongest deviations for signatures with highest evidence (Figure S4 in File S1). The sensitivity is also slightly better using the spherical transformation as evaluated by the increased number of signatures with significant association. The results of the GSEA-based implementation are similar to the results without spherical transformation, reflecting a superiority of the presented method (Figure S4 in File S1).

The almost monotonic association between PhysioScores and permutation p-values suggests that the PhysioScore is a valid measure to rank the signatures according to their significance, even though it is not possible to determine a rigorous significance threshold. This result is very important for applications were the number of replications is too low for sample label permutation.

### Tracking differentiation time series

In vitro differentiation of pluripotent stem cells into diverse somatic cell types is increasingly studied in order to obtain a molecular understanding of embryogenesis, to build disease specific in vitro models, and to develop new options for regenerative medicine and drug development [34-36]. An important task in this context is the detailed characterization of cell identity and differentiation dynamics. The molecular changes during differentiation are usually monitored based on the expression of a few cell type specific marker genes. The PhysioSpace method in contrast is capable of characterizing the phenotypic changes in the context of large scale gene expression patterns, thus complementing the routinely performed marker gene based analyses. 

We mapped the dynamic changes of three in vitro differentiation time series (table 4) into the PhysioSpace. A neural differentiation (GSE9940) with expression data of H9 embryonic stem cells (ESCs, day 0), embryoid bodies (EBs) at day 6 of differentiation as well as primitive neural epithelial cells at days 10 and 17 of differentiation. A cardiac differentiation (GSE28191) with six time points (day 0, 2, 5, 7, 9, and 11) and a trophoblast differentiation time series (GSE30915) incorporating seven time points (day 0, 2, 4, 6, 8, 10, and 12). In all cases, the gene expression of differentiating cells was compared to the starting undifferentiated ESCs. A rigorous statistical validation is not possible, due to the small number of sample replicates in each time step. However, according to the previous results, the PhysioScores provide valuable information, that can be derived from a ranking of PhysioSpace signatures.

**Table 4:** Differentiation time series datasets.

Accession	Usage	Description
GSE9940	Neural differentiation	18 samples from a differentiation of ESCs towards neural precursors at day 0 (3), day 6 (3), day 10 (6), or day 17 (6)
GSE30915	Trophoblast differentiation	21 samples from a differentiation of ESCs towards trophoblasts at days 0, 2, 4, 6, 8, 10, and 12 with 3 samples per time point
GSE28191	Cardiac differentiation	12 samples from a differentiation of ESCs towards cardiomyocytes at days 0, 2, 5, 7, 9, and 11 with 2 samples per time point
GSE10469	Additional trophoblast differentiation	2 trophoblast differentiation time series with 4% or 20% O2 at 0, 3, 12, 24, 72, and 120 hours of differentiation

**Figure 6:**
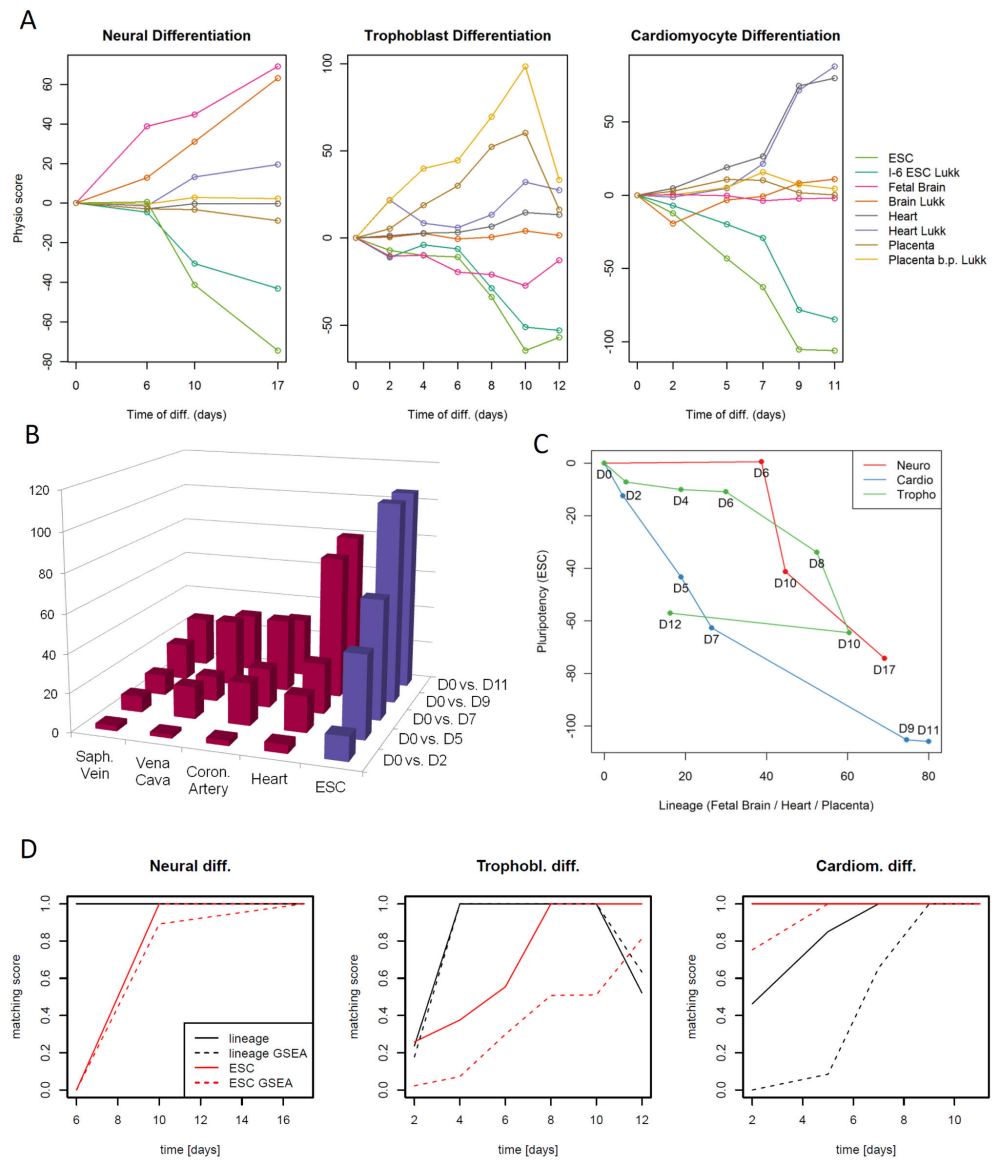
Detailed results of the differentiation time series analyses. (A) Line plots of most relevant PhysioScores for the three differentiation time series comparing scores from PhysioSpaces 1 and 3. Lines with names ending with “Lukk” correspond to the third PhysioSpace (Lukk et al. 2010 [18], E-MTAB-62), other lines correspond to the first PhysioSpace (GSE7307). (B) Heart and ESC increasingly dominate leading PhysioScores in the cardiomyocyte differentiation time series. Depicted are the PhysioScores of the 5 strongest signatures from cardiomyocyte differentiation. Red (blue) colors correspond to positive (negative) PhysioScores. (C) Comparison of pluripotency (ESC) and lineage (fetal brain/placenta/heart) scores for the three differentiation time series exhibit different dynamics in PhysioSpace 1. The lineage score corresponds to the dominant lineage in each differentiation, i.e. fetal brain, placenta, and heart for the neural, trophoblast, and cardiomyocyte differentiation, respectively. (D) The matching score is used to compare the implemented PhysioSpace algorithm to a classical GSEA based algorithm, showing relatively strong differences between the two methods for the trophoblast and cardiomyocyte differentiations. The implemented PhysioSpace algorithm has generally a higher matching score.

In a global perspective, the a priori expected signatures dominate over time (Figure 5). The ESC PhysioScore gradually decreases, i.e. becomes more negative with continuing differentiation, while the lineage-specific PhysioScores, i.e. fetal brain, placenta, and heart increase for the neural, trophoblast, and cardiomyocyte differentiations, respectively (Figure 5 and Figure 6 A-C). The sole exception is day 12 in the trophoblast differentiation, showing a sharply decreasing placenta-score compared to day 10 (Figure 6 A, C). In the publication going along with the trophoblast differentiation dataset [37] microarray data are only described up to day 10 of differentiation. In order to investigate the sharp decrease in the placenta score from day 10 to day 12, several trophoblast related genes that were identified to be strongly upregulated from day 0 to day 10 of differentiation [37] were analyzed. We identified several genes that show a similarly sharp decrease from day 10 to day 12 of differentiation, including PGF, GCM1, GATA2, GATA3, MFAP5, KRT7, KRT8, MUC15, and HSD3B1, which were all described to be trophoblast related [37] (Figure S8 in File S1). Other trophoblast related genes do not show such a sharp decrease, including CGA, CGB, and ID2. These results indicate that the PhysioScore captures a real biological phenomenon which would not have been detected in single marker analysis of CGA, CGB, or ID2 genes.

For the neural differentiation, a consistently increasing PhysioScore was observed for the whole cluster of neural tissues (Figure 5). However, the Fetal Brain score is clearly dominating, indicating that the differentiated neural epithelial cells are more similar to fetal brain than to adult brain. Another interesting observation is the rather high fetal brain score and the negligible decrease in the ESC score for the differential expression between ESCs (day 0) and EBs (day 6) (Figure 6 A, C). This could be interpreted in such a way that, although a neural transcriptional program is strongly induced in ESC under neural differentiation conditions after 6 days, a significant amount of residual pluripotent stem cells remain in the culture at day 6 and still have to differentiate in order to render the neuralized cells safe, e.g. for cell therapy purposes.

Looking closer into the cardiomyocyte differentiation, a striking increase of the heart score can be observed from day 7 to day 9 of differentiation (Figure 6 A-C). Up to day 7, the heart score is fairly similar to other scores representing cardiovascular tissues, like the Coronary artery or vena cava (Figure 6 B). The sharp increase from day 7 to day 9, suggestive of cells passing a sharp decision point, clearly distinguishes the heart score from the others (Figure 6 B). Therefore, it would be interesting to analyze the differentiation around day 7 more closely in future studies in order to detect the molecular mechanisms behind this striking change.

In Figure 6 C the dynamical changes of the pluripotency (ESC) score and the lineage score (fetal brain, placenta, or heart) are depicted. Comparing the three differentiation time series to each other, a difference in the dynamics was detected. The neural and trophoblast differentiations show an increase in the lineage score and a fairly constant ESC score up to day 6 of differentiation. The ESC score decreases mainly after day 6. For the cardiomyocyte differentiation, in contrast, a sharp decrease of the ESC score can already be observed in early differentiation steps, and the lineage score has its strongest increase after day 7.

The three differentiation time series were also used to compare the presented algorithm to the GSEA-based implementation. The matching score, as used in the simulation results, was calculated for each time point (Figure 6 D, Figure S9 in File S1), showing a better matching performance of our algorithm. The main differences can be observed for the ESC score in the trophoblast differentiation as well as the heart score in early time points of the cardiomyocyte differentiation.

The robustness of the PhysioSpace method was evaluated by constructing three different PhysioSpaces. For this, we have used the same construction approach, yet employed three different gene expression data collections, which contained similar biological phenotypes (Materials and Methods). The embedding of the three differentiation time series and three cancer datasets into these three separate PhysioSpaces are depicted in Figures S5, S6, and S7 in File S1, showing comparable overall results among those three independently constructed PhysioSpaces. The results for the third PhysioSpace (Lukk et al, E-MTAB-62) are slightly more arduous to interpret due to the large size of this particular PhysioSpace (> 5000 microarrays) which combines several studies from different laboratories with sometimes inconsistent annotations. Nevertheless, the dominating scores induced by the three exemplary differentiation time series are still scores corresponding to brain, placenta, or heart, consistent with a-priory expectations and the results of the main PhysioSpace.

For future extensions of the PhysioSpace the possibility to combine signatures from different microarray platforms is going to increase the universal applicability and thus utility of the PhysioSpace approach. In order to be comparable, the absolute values of the PhysioScores must be in a similar range. We tested the principal possibility of a combination by comparing the PhysioScores from the three different PhysioSpace constructions. A linear adjustment according to the number of matching probes between the signature and the dataset was applied to approximately account for the resulting different gene-set sizes (Materials and Methods). A selection of the most prominent signatures from all three PhysioSpace constructions is depicted in Figure 5. The color-scheme is kept constant for all three PhysioSpaces to allow comparison. The signatures of PhysioSpaces 1 (GSE7307) and 3 (Lukk et al, E-MTAB-62) are in similar ranges (Figure 6 A), PhysioSpace 2 (GSE2361) has slightly lower absolute values (Figure 5). In conclusion a combination of different PhysioSpaces is possible, at least in the case were a compact graphical representation allows a visual comparison across different spaces.

In order to additionally test the robustness and platform independence of the PhysioSpace method, we analyzed an additional trophoblast differentiation time series (GSE10469, Agilent-014850 Whole Human Genome Microarray 4x44K G4112F microarray platform). The differentiation was performed in two settings with either 4% or 20% oxygen. In both settings, the Placenta-score and ESC-score dominate (Figure S3 in File S1), supporting the validity of the method for cross-platform analyses.

## Discussion

A robust signature association method was developed that allows the linkage of data from cellular assays with clinical data and outcomes. This approach enables the contextual interpretation of newly performed experiments in a physiological “space” as represented by signatures generated from publicly available microarray data sets, allowing “big data” approaches. The signatures can be interpreted as directions in a high dimensional gene expression space. Following this geometrical interpretation, the PhysioSpace represents a low dimensional subspace of the space spanned by all gene expression patterns, having all a physiological interpretation. Differential expression vectors from newly performed experiments are compared to the reference signatures by robust methods extending gene set enrichment algorithms to account for the noise and heterogeneity in the data. The comparison is performed on differential expression patterns rather than absolute expression values in order to avoid the direct comparison of absolute gene expression values from heterogeneous protocols.

The combined use of gene permutation and sample-label permutation together with the robust signature ranking ability of the PhysioScores allows a detailed and valid statistical interpretation of the results. It is possible to use the PhysioScore values for ranking purposes in cases were sample permutation is not feasible, e.g. if a class contains less than 7 samples. In this case the relevance of the high ranking features has to be supported by other means, such as additional experiments and analyses, literature, or marker genes. The Prostate Cancer example shows that the sample-label permutation approach avoids false positives, helping to decide on further investigations and possibly saving resources.

A spherical transformation has been shown to improve the concordance of the PhysioScore with permutation derived significance assessment. Since the PhysioSpace method compares only directions of vectors rather than their lengths, sample label permutation for elliptical distributed data creates many vectors with similar directions and, hence, similar PhysioScores (Figure 2 B). The spherical transformation diminishes this effect by reduction of ellipticity in the data (Figure 2 C). On one hand, the transformation is necessary to reduce the possibly strong influence of non-phenotype associated variation on the definition of gene sets in the analyzed data. On the other hand, it is necessary to create a meaningful null distribution in the sample-label permutation approach.

Classical gene set enrichment approaches use predefined gene sets and evaluate the enrichment of these sets on the differential expression in the data under investigation. In contrast, the method presented here defines the gene sets using the new data and calculates the enrichment on the PhysioSpace signatures, essentially implementing a backward gene set enrichment approach. This gives a slightly different perspective on the data since the strict cutoff of genes is performed on the new data instead of the retrospective data. In addition, there is also a difference from a statistical point of view. Many biological studies do not have more than three to five independent replicates, making sample permutation infeasible. Especially for time series experiments the relatively large amounts of replicates (at least seven to nine samples) needed for the sample permutation approach can become very cost intensive. Therefore, many studies rely on gene label permutation, which is not sufficient for rigorous assessment of statistical significance, bearing the risk of “heading down the wrong pathway” [33]. The reason for this is the correlation structure within gene sets, violating the independence assumption made by most statistical tests. However, while gene label permutation cannot be used for rigorous significance assessment, it is valid for ranking of gene sets or signatures in order of decreasing significance, as long as the correlation structure and gene set size is similar among gene sets. The backward direction of gene set enrichment, implemented in the present article, defines the gene sets based on the new data. Thus, the gene sets, and consequently their correlation structures, are identical for the enrichment calculation of each signature. This results in a valid ranking of signatures according to their significance, even in the case of very low sample sizes.

Three different types of cancer data were analyzed in order to show the performance of the PhysioSpace method. The results show different kinds of outcome with many, few, or no significant signature associations for breast, lung and prostate cancer datasets. It is already known that determination of biomarkers depends not only on the size of studies but primary on the clinical phenotype [38,39]. A biomarker may be developed with nearly any gene expression signature, as in the case of breast cancer grade [16], or only with a very specific signature, if at all, as in the case of prostate cancer Gleason scores [40,41]. The PhysioSpace method allows a stable ranking of different signatures, indicating which signatures may be most appropriate for biomarker discovery. The sample-label permutation approach can then be used to determine the significance of the association, evaluating whether or not the results can be replicated with new data drawn from the same population.

The detailed characterization of human pluripotent stem cells as well as their differentiation dynamics is important for quality control and understanding of the mechanisms and dynamics of cell fate changes. Besides the analysis of single marker genes and proteins, a whole genome based characterization can provide more robust information and outcome measures [6,7]. Large scale pattern based characterization of pluripotent stem cells (iPSC) has been developed [6,42]. However, for in vitro differentiated cells, large scale gene expression based quality control is complicated due to the need to compare in vitro cultivated cells to cells from tissues, showing widely differing gene expression [19]. The PhysioSpace method allows determining the direction of differentiation. Thus, it can be evaluated whether the cells differentiate along the designated lineage. The determination of directions rather than absolute locations with the proposed robust statistical methods allows the application of the PhysioSpace method across microarray platforms. For absolute localization, in contrast, it is usually necessary to specify a reference set for each microarray platform [43,44]. In addition to characterizing differentiated cells, it is also possible to gain insight into differentiation dynamics. The analysis of differentiation time series data allows to unravel time points with unexpected dynamics. For example, the analysis of the cardiomyocyte differentiation suggests a potential “breakpoint” in the heart-scores around day 7 of differentiation (Figure 6 A-C), whereas the dynamics of the pluripotency-related scores show an almost linear decrease, indicating the existence of cell-fate decision points along the differentiation pathway. This insight could be used to direct further investigations into the differentiation mechanisms at work around these special time-points which may be critical for the overall dynamics of the underlying biological process. 

The PhysioSpace compendium is derived from publicly available gene expression data in a straightforward and resource effective manner. More specific compendia for specific applications can be derived easily from the large amount of available datasets.

The robustness of the PhysioSpace method allows using very small number of replicates, reducing experimental efforts at the same precision. The high biological relevance of the results confirm its usefulness for wide ranges of applications in drug discovery and (trans-) differentiation approaches. It allows to utilize the tremendously growing repositories of existing data for interpretation of specific wet-lab experiments on the background of physiology, possibly establishing a quantitative link between lab experiments and clinical applications. 

RNA-seq and related next-generation technologies have an enormous potential for high resolution measurements of small cell populations. Properties of specific measurement techniques must be considered, when applying the PhysioSpace method to datasets created by different measurement techniques. Processing pipelines and normalization for next-generation sequencing data is an active field of development and all count based methods introduce bias through low counts and different transcript lengths [45] that cannot be removed by normalization alone, but needs to be addressed by the statistical method [46]. Methods such as PhysioSpace, that are designed with a focus on robustness and rigorous statistical evaluation, can provide a starting point to integrate results from new data sources and promote biological insights in future applications. 

## Materials and Methods

### Microarray data acquisition and preprocessing

All datasets were downloaded from Gene Expression Omnibus (GEO, http://www.ncbi.nlm.nih.gov/geo/) [47] or ArrayExpress (http://www.ebi.ac.uk/arrayexpress) [48] databases. Either preprocessed data were taken (GSE2361, E-MTAB-62, GSE2990, GSE10072, GSE10469, GSE16560, GSE33789, GSE19804, GSE21034 – transcript version) or the data were preprocessed using the apt-probeset-summarize method of the Affymetrix Power Tools software package (Affymetrix Power tools. http://www.affymetrix.com/partners_programs/programs/developer/tools/powertools.affx. Accessed 2013 September 16.) with RMA normalization (GSE7307, GSE23402, GSE9940, GSE30915, GSE28191, GSE18676).

Probe identifiers of different microarray platforms were matched using the getBM method of the biomaRt R package [49].

The breast cancer analyses were performed on the GSE2990 dataset using all data that have information on breast cancer grade. No distinction according to estrogen receptor status was made. Analyses were performed for comparison of breast cancer grade 1 vs. 2 and grade 1 vs. 3.

Dataset GSE10072 was used for the analysis of lung gene expression of never vs. former and never vs. current smokers using all samples from cancerous and adjacent tissues in a single analysis. For the investigation of prostate cancer all primary tumors with Gleason-score 6, 7, 8, or 9 from dataset GSE21034 (transcript version) were used. Comparisons were made between samples of Gleason score 6 vs. 7, 6 vs. 8, and 6 vs. 9. In a separate analysis, primary tumors were compared to metastases. Additionally, all data from dataset GSE16560 were used to analyze differences associated with Gleason scores.

For the neural, trophoblast, and cardiomyocyte differentiation analyses, datasets GSE9940, GSE30915, and GSE28191 were used, respectively. Samples were grouped according to time of differentiation. No distinction according to treatment was made for the neural differentiation. An additional trophoblast differentiation dataset (GSE10469) was analyzed with separate analyses for the normoxic (20% oxygen) and the hypoxic (4% oxygen) conditions. In all comparisons, differentiating cells were compared to the starting pluripotent stem cell samples.

### Simulation of mixed samples

The computational mixing was achieved by a linear combination of tissue and ESC samples with mixing factor *λ*

$$x_{mixed}=\lambda x_{ESC}+(1-\lambda )x_{tissue}$$

The mixing was performed on non-transformed data, in contrast to all other calculations that were performed on log_2_-transformed data. For the first simulation, *x*_*ESC*_represents a dataset with 20 ESC samples, obtained from GSE33789 by taking each ESC sample twice, and *x*_*Lung*_the first 20 of the 40 randomly drawn lung samples, obtained from GSE19804. Hence, the data simulate an infiltration of up to 5% ESCs (*λ*=0,0.01,…,0.05) into the rather heterogeneous samples from lung cancer and adjacent lung tissue. In the second simulation, the first 5 ESC samples from GSE33789, each taken twice, were mixed with 10 samples of adjacent normal lung tissue (GSE19804) and compared to the remaining 5 ESC samples. The tissue fraction in the mixture ranges from 0.1 to 1 in steps of 0.1, i.e. the fraction of ESCs was*λ*=0.9,0.8,...,0. For the third simulation, a single ESC sample from dataset GSE33789 was mixed with each of the 22 tissues and 2 cell lines from dataset GSE18676 and compared to the remaining 9 ESC samples. The mixing factor was set to*λ*=0.97.

### PhysioSpace generation

The PhysioSpace consists of a compendium of gene expression signatures, representing vectors of differential expression. Differential expression is calculated using a Student’s t-test between samples from a specific tissue or cell line and a computationally built reference. The reference is chosen as the vector of mean expression values of all samples in the dataset, in order to simulate a common reference showing no tissue-specific expression. The standard error of the mean expression is used as standard deviation of the reference for calculation of t-tests. The signed log_10_-p-values of the t-tests are used as PhysioSpace signatures.

Three different PhysioSpaces were built for the analyses to show the robustness of the presented results (table 1). The first PhysioSpace consists of a total of 94 signatures built from datasets GSE7307 and the embryonic stem cell (ESC) data from GSE23402. The assignment of samples to signatures was done according to the sample annotations. The 94 signatures were calculated on the 18484 probes that could be associated with a hgnc-symbol and are present on both the Affymetrix GeneChip Human Gene 1.0 st and the Affymetrix GeneChip Human Genome U133 Plus 2.0 arrays. 

The second PhysioSpace was built based on the GSE2361 dataset using all available probes. This dataset consists of 36 samples, each representing a different human tissue. Thus, the second PhysioSpace consists of 36 signatures, each representing the differential expression of a single sample to the computationally built common reference.

The 369 signatures of the third PhysioSpace were built according to the 369 Groups annotated in dataset E-MTAB-62. Again, all probes of the Affymetrix GeneChip Human Genome U133A array were used to calculate the signatures.

For visualization purposes the signatures were hierarchically clustered based on a Pearson-correlation distance using average-linkage, indicating some common gene expression features of signatures clustering closely together.

### Mapping of gene expression differences onto the PhysioSpace

For a comparison of two phenotypes, e.g. different times of differentiation, or cancer stages, the mapping of the differences in gene expression onto the PhysioSpace is done by the following three-step procedure consisting of a spherical transformation, a data-based definition of gene sets and an enrichment calculation via a Wilcoxon rank-sum test.

#### Spherical transformation

The spherical transformation of the data is an essential step for the statistical validation. It allows the meaningful calculation of a null-distribution of the enrichment score via sample label permutation (Figure 2 B, C, Figure 3 B). 

Starting from a gene-wise standardized (i.e. centered and scaled) data matrix *D* a singular value decomposition is calculated (*D*=*UΣV* ^*T*^). The entries of the diagonal matrix *Σ* are then truncated to a maximum of *t* (${\tilde{\Sigma }}_{ij}=\min(\Sigma_{ij},t)$), what can be interpreted as a normalization of the principal components. In a final step, the spherically transformed data are calculated as$\tilde{D}=U\tilde{\Sigma }V^{T}$. The spherical transformation is applied pair-wise, i.e. for each comparison of two phenotypes. In the application examples the truncation parameter *t* was set to 1.

#### Data based gene set definition

In the second step of the mapping procedure two sets of up- and down-regulated genes are determined, each containing the top 5% of up- and down-regulated genes, respectively. Genes are ranked corresponding to their mean fold changes between the two phenotypes. 

The cutoff of 5% up- or down-regulated genes was chosen, on one hand, to focus on large scale patterns rather than a few genes and, on the other hand, to exclude genes that are only driven by noise. In the application examples it turned out that the results were quite robust with respect to a variation of the cutoff parameter between 1% and 10% (data not shown).

#### Rank sum based enrichment score

In the third step, a gene set enrichment score is calculated on the PhysioSpace signatures using the gene-sets of the previous step. The wilcox.test procedure of the R stats-package [50] is used to calculate a Wilcoxon rank-sum test between the sets of up- and down-regulated genes. The final enrichment score (called PhysioScore) is then defined as the signed log_10_ p-value of the Wilcoxon-test. 

### Statistical validation

A sample-label permutation approach with B=1000 permutations is used to rigorously determine statistically significant signatures. The permutation p-value is defined as 

$$\frac{1+\sum_{b=1}^{B}I(s_{b}\geq s_{0})}{1+B}$$

where *s*_0_ is the absolute value of the observed PhysioScore, *s*_*b*_,*b*=1,…,*B*, are the absolute values of the permutation PhysioScores, and *I* is the indicator function, being 0 if s_b_ is smaller than s_0_ and 1 otherwise. All sample permutation p-values were adjusted for multiple testing using the Benjamini-Hochberg correction [32].

### Scaling of PhysioScores for inter-platform comparisons

In Figure 5 and Figure 6 A, PhysioScores corresponding to gene expression signatures from different microarray platforms are compared. Due to the different platform designs there exist some differences in the number of probes between platforms resulting in different numbers of genes used for the Wilcoxon test. Assuming independence of platform design and gene expression differences in the data, i.e. values are missing at random; the effect of the different sample size on the log_10_ p-value is approximately linear in the relevant range (data not shown). Therefore, the PhysioScore values were linearly transformed to simulate same number of genes. 

### Implementation and availability

All analyses were conducted in the R programming language [50], R version 2.13.0 under Windows. The R codes for the spherical transformation and the mapping of new data into the PhysioSpace are available as supplemental material (File S3). The three PhysioSpaces implemented and discussed in the manuscript are available upon request. The PhysioScores and permutation p-values of the differentiation time series and cancer analyses are available as supplemental excel file (File S2).

## Supporting Information

File S1 **Combined supporting information file of additional figures.** Figure S1. Self-similarity heatmap of PhysioSpace 1. Figure S2. PhysioScores for the first simulation scenario. Figure S3. PhysioScores of additional datasets. Figure S4. Comparison of PhysioScores and permutation p-values. Figure S5. Extension of Figure 5 showing the entire PhysioSpace 1. Figure S6. Extension of Figure 5 showing the entire PhysioSpace 2. Figure S7. Extension of Figure 5 showing the entire PhysioSpace 3. Figure S8. Single gene analysis of Trophoblast differentiation. Figure S9. Heatmap comparing the differentiation time series results from the presented method to the GSEA-based implementation.
(PDF)

File S2 **Excel file of PhysioScores and permutation p-values of all datasets from the main analyses.**
(XLSX)

File S3 **R-script for the calculation of PhysioScores and permutation p-values.**
(TXT)
