# Supplementary material for: PhysioSpace: Relating Gene Expression Experiments from Heterogeneous Sources Using Shared Physiological Processes
Source: PLoS One. 2013 Oct 17;8(10):e77627. doi: 10.1371/journal.pone.0077627 (PMC3798402; doi:10.1371/journal.pone.0077627)
Supplement: File S1 — Combined supporting information file of additional figures. Figure S1. Self-similarity heatmap of PhysioSpace 1. Figure S2. PhysioScores for the first simulation scenario. Figure S3. PhysioScores of additional datasets. Figure S4. Comparison of PhysioScores and permutation p-values. Figure S5. Extension of Figure 5 showing the entire PhysioSpace 1. Figure S6. Extension of Figure 5 showing the entire PhysioSpace 2. Figure S7. Extension of Figure 5 showing the entire PhysioSpace 3. Figure S8. Single gene analysis of Trophoblast differentiation. Figure S9. Heatmap comparing the differentiation time series results from the presented method to the GSEA-based implementation. (PDF) [file pone.0077627.s001.pdf]

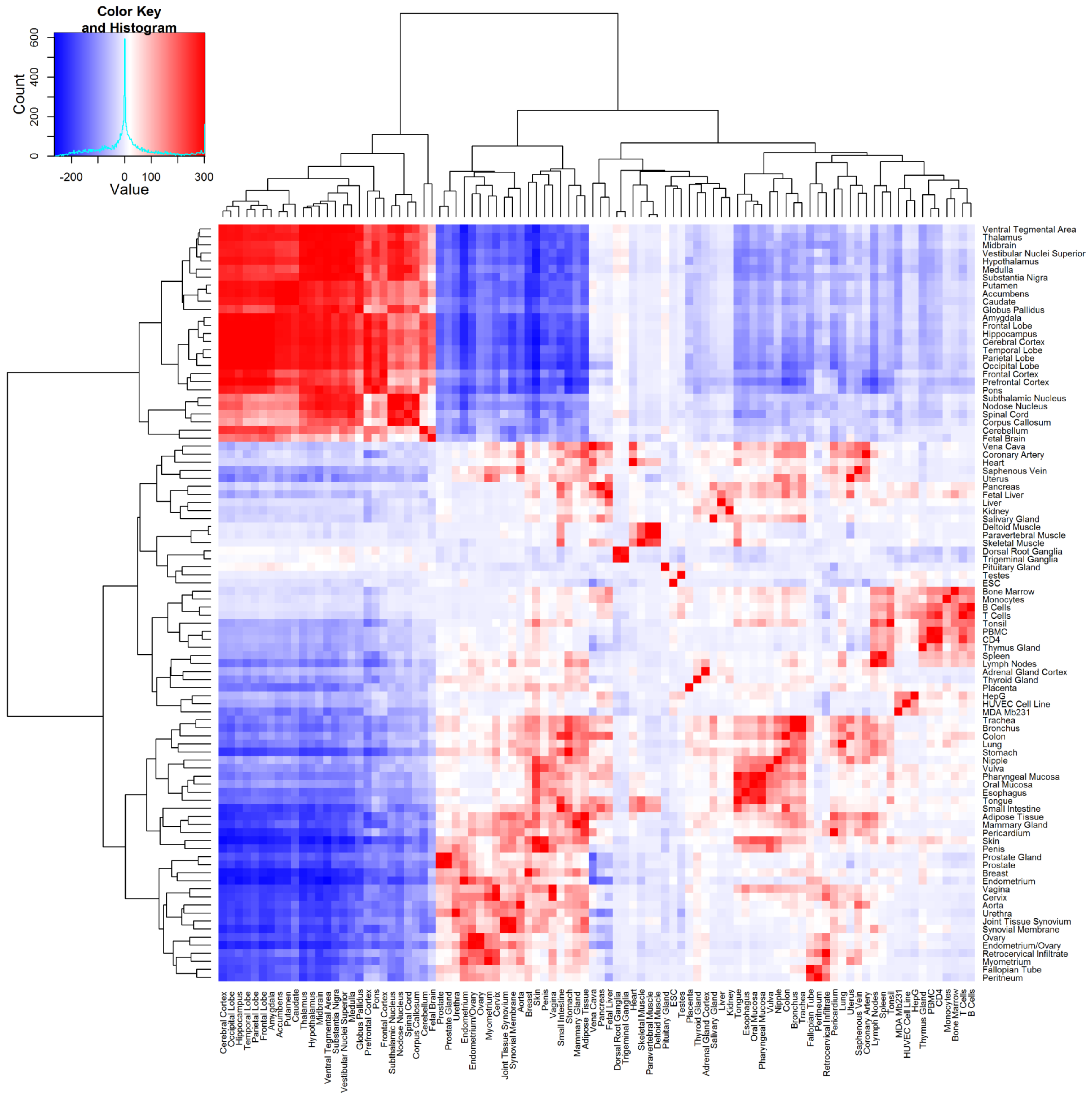

Figure S1 Self-similarity heatmap of PhysioSpace 1. A comparison of the PhysioSpace signatures to each other via the proposed mapping procedure highlights the non-orthogonality of the signature directions. Tissues involving cells with similar function show high correlations. Most tissues consist of several cell types and some cell types are present in many tissues, e.g. fibroblasts. The asymmetry in the heatmap is due to the gene set enrichment approach used in the mapping procedure, focusing on genes with highest differential expression in the dataset under investigation.

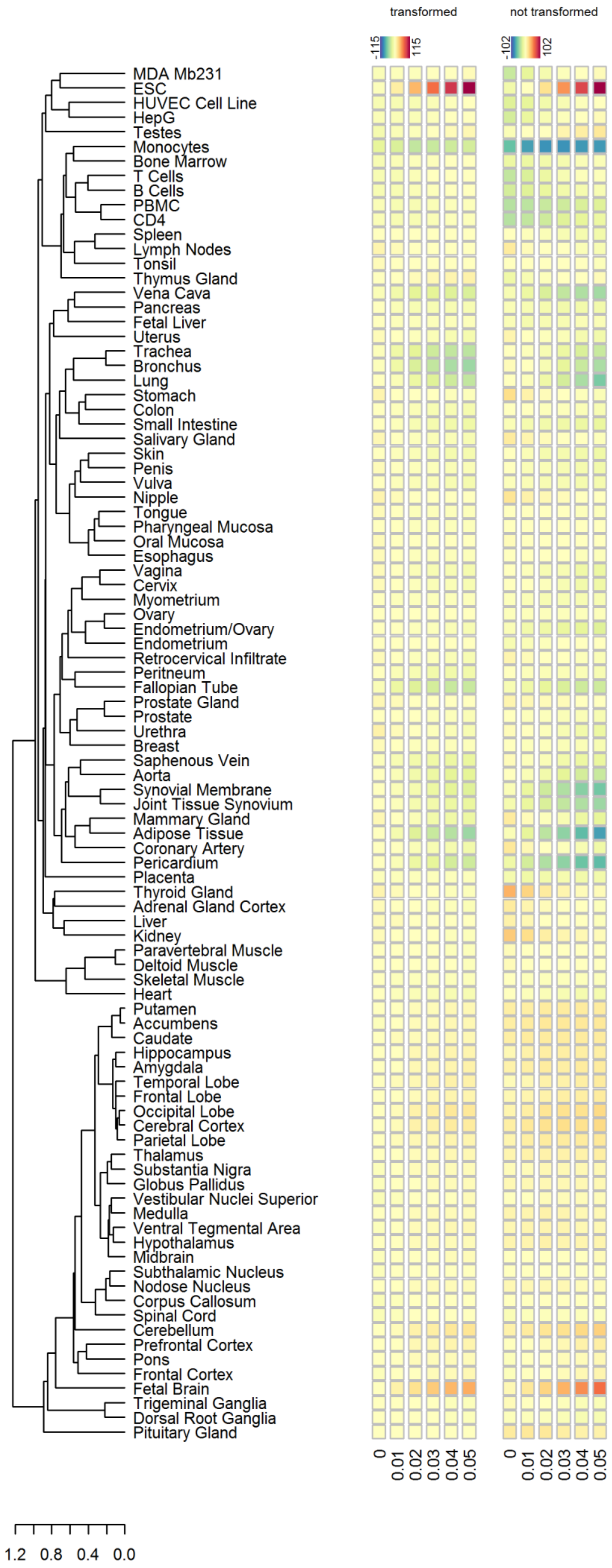

Figure S2 PhysioScores for the first simulation scenario. Extension of Figure 3 A showing the entire PhyisoSpace 1. The spherical transformation decreases non-phenotype associated heterogeneity leading to more sensitive and specific results.

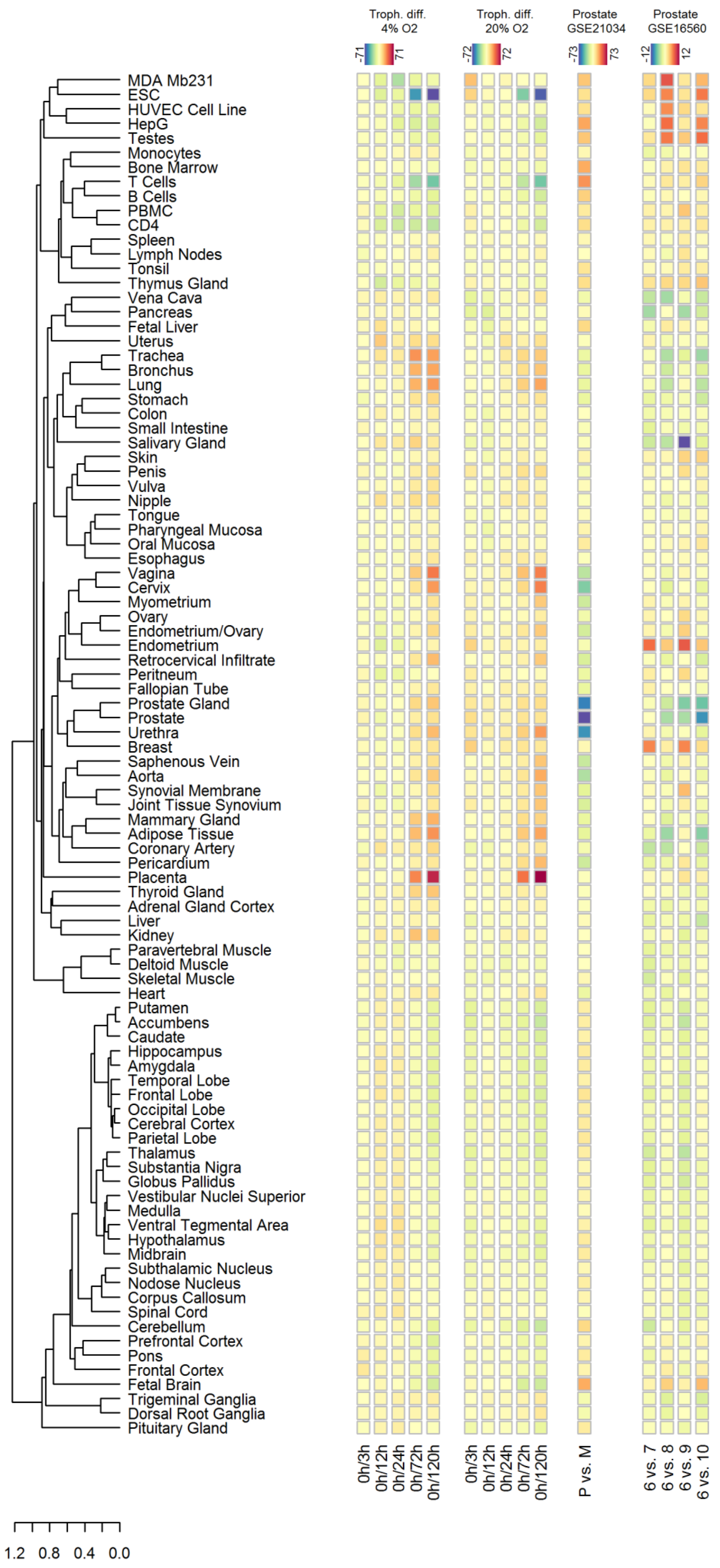

Figure S3 PhysioScores of additional datasets.
Heatmap of PhysioScores from the first PhysioSpace (GSE7307, GSE23401) analyzing two trophoblast differentiation time series (GSE10469), and two cancer data sets (GSE21034, GSE16560). The trophoblast differentiation time series were hybridized on an Agilent array, showing the crossplatform performance of the PhysioSpace method. The initial ESCs (0h of differentiation) were compared to differentiating cells (3h, 12h, 24h, 72h, and 120h of differentiation). For dataset GSE21034, primary prostate tumor samples were compared to metastases. Association to Prostate is strongly negative, indicating that metastases are less similar to prostate than primary prostate tumors, as expected. Some immune and cell line scores are increased for metastases. An increased Gleason score in prostate tumors shows a weak association with ESC and cell line scores in dataset GSE16560. A similar association could not be found in primary tumors of dataset GSE21034 (Figure 5).

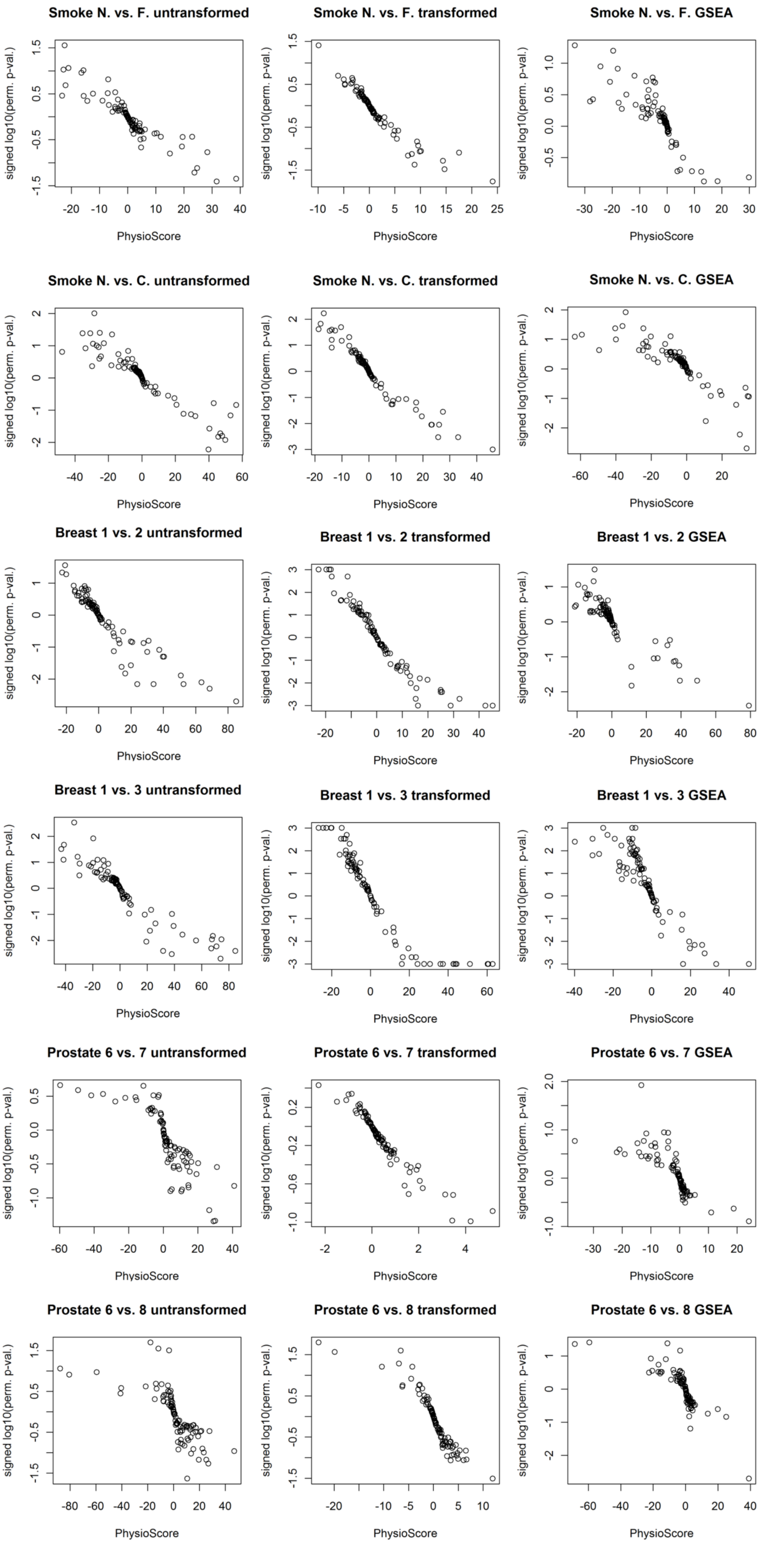

Figure S4 Comparison of PhysioScores and permutation p-values.
PhysioScores are plotted versus signed $\log_{10}$ sample permutation p-values for the cancer datasets. The results indicate that the spherical transformation leads to a more monotonous relation between the two quantities (first and second column). The GSEA-based implementation leads to worse results than the implemented algorithm, i.e. has a less monotonous relation (third column). The permutation p-values are slightly increased when the transformation is used, resulting in an increased sensitivity.

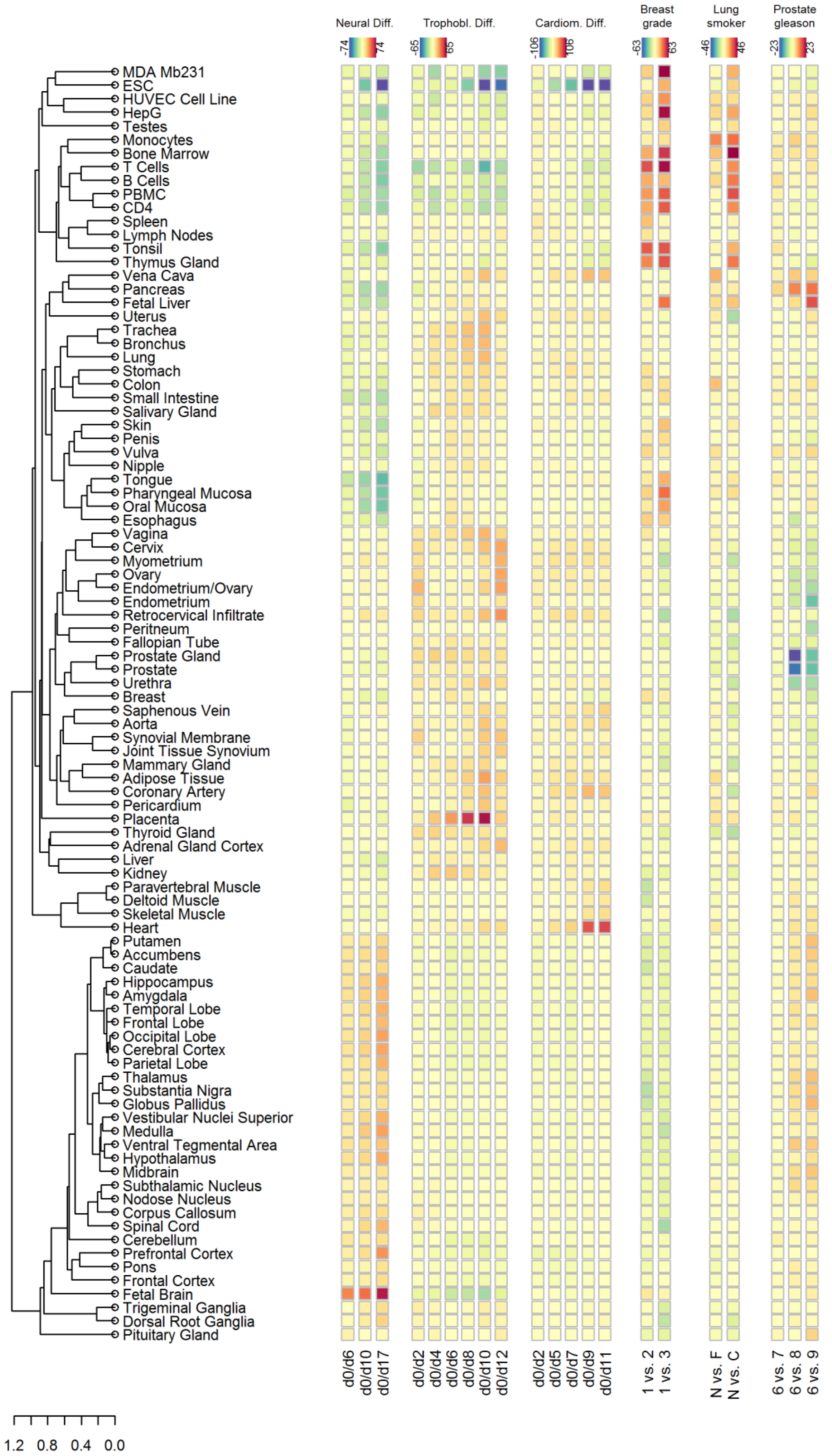

Figure S5 Extension of Figure 5 showing the entire PhysioSpace 1.
PhysioScores of the three timeseries (GSE9940, GSE30915, and GSE28191) and three cancer datasets (GSE2990, GSE10072, and GSE21034) are visualized in a heatmap-like representation. The 94 signatures of PhysioSpace 1 are clustered according to a Pearson-correlation distance at the left hand side. PhysioScores are color-coded with colors ranging from negative values in blue and green to positive values in orange and red. The absolute values differ between datasets as indicated in the colorbars. Timeseries results show dominating neural, trophoblast, or heart signatures, increasing with differentiation as well as a decreasing ESC signature. Immune and cell line signatures dominate the cancer results.

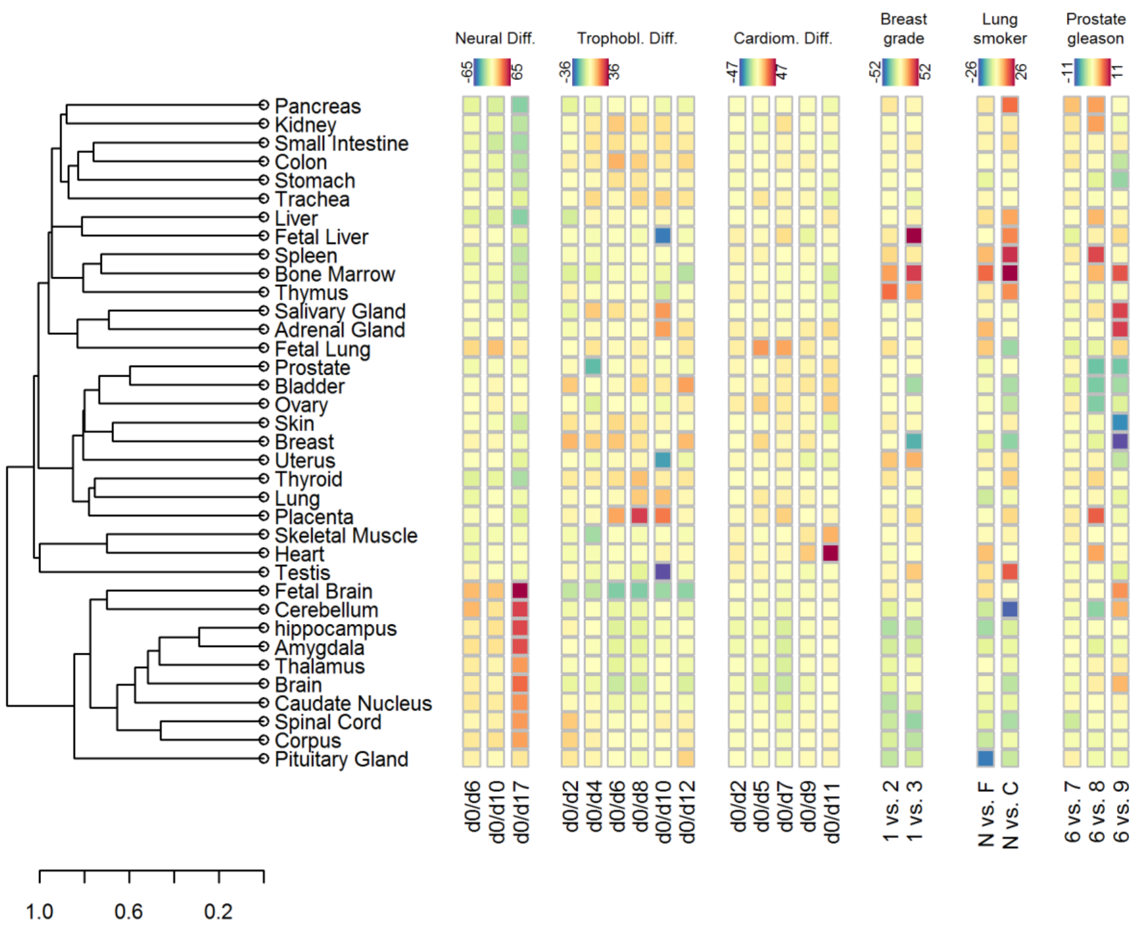

Figure S6 Extension of Figure 5 showing the entire PhysioSpace 2.
PhysioScores of the three timeseries and three cancer datasets are visualized in a heatmap-like representation with hierarchically clustered signatures on the left and color-coded PhysioScores on the right. Timeseries are dominated by the expected signatures (neural, placenta, heart). There is no ESC signature within the 36 signatures of PhysioSpace 2. Breast and lung datasets are dominated by immune signatures. Additionally, the fetal liver signature has a high PhysioScore for the comparison of breast cancer grade 1 to grade 3. Gleason scores in prostate cancer have relatively low PhysioScores. Sample label permutation results in no significant association (data not show).

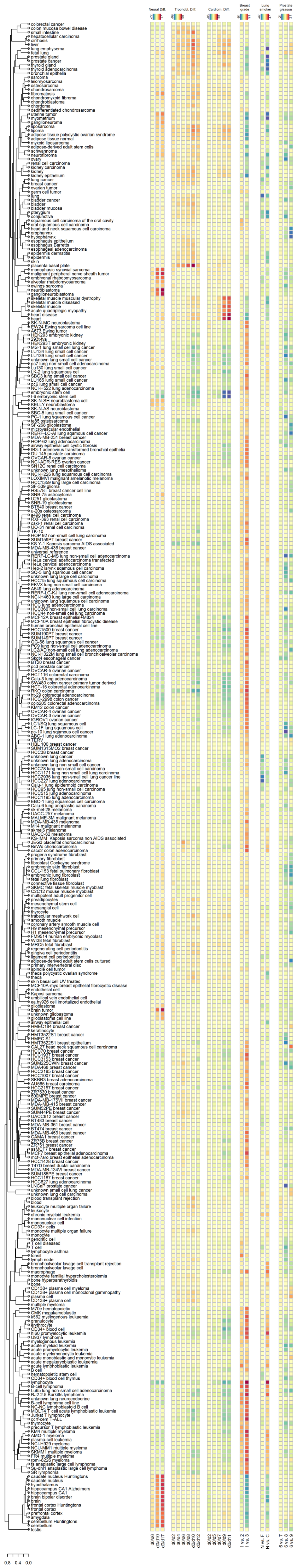

Figure S7 Extension of Figure 5 showing the entire PhysioSpace 3.
PhysioSpace 3 incorporates 369 signatures that were generated from a large dataset (E-MTAB-62) incorporating data from many different studies. The dataset seems to be quite heterogeneous leading to a sometimes counter-intuitive hierarchical clustering of the signatures. Despite this heterogeneity, the dominating signatures are overall in accordance with the results of the other PhysioSpaces. Differentiation time series are dominated by neural, placenta and heart/muscle scores and show a decreasing ESC score. Breast cancer grade is associated with many fast proliferating signatures, e.g. cancer signatures, as well as immune signatures. Lung samples of smokers show increased immune signatures. Differences between samples with differing prostate cancer Gleason scores have comparably low PhysioScores.

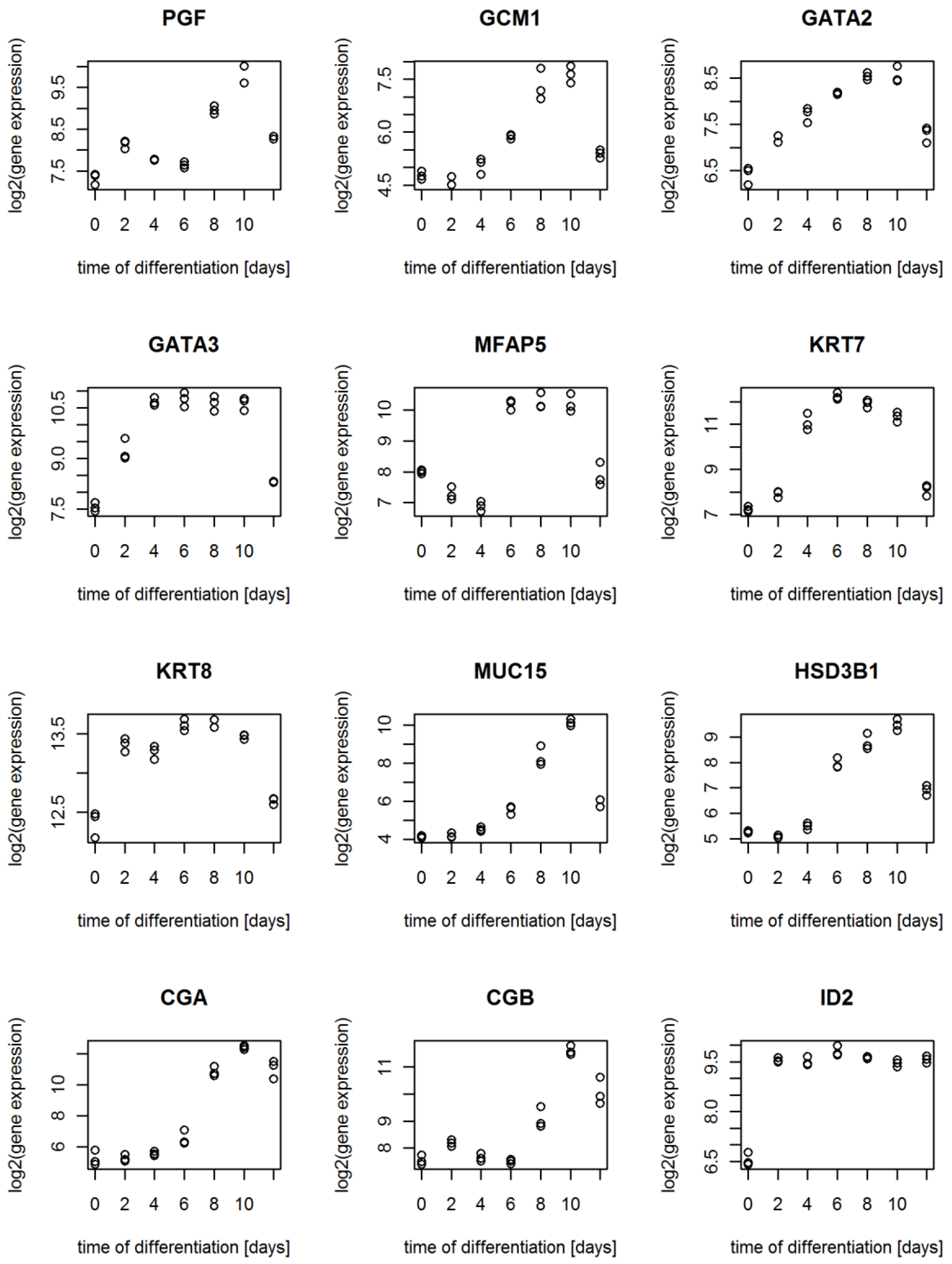

Figure S8 Single gene analysis of Trophoblast differentiation.
Analysis of several genes with known trophoblast association shows an increase of gene expression up to day 10 of differentiation and a subsequent decrease (PGF, GCM1, GATA2, GATA3, MFAP5, KRT7, KRT8, MUC15, HSD3B1). A similar pattern is present in the PhysioSpace analyses. Some other trophoblast associated genes decrease only moderately or not at all from day 10 to day 12 (CGA, CGB, ID2).

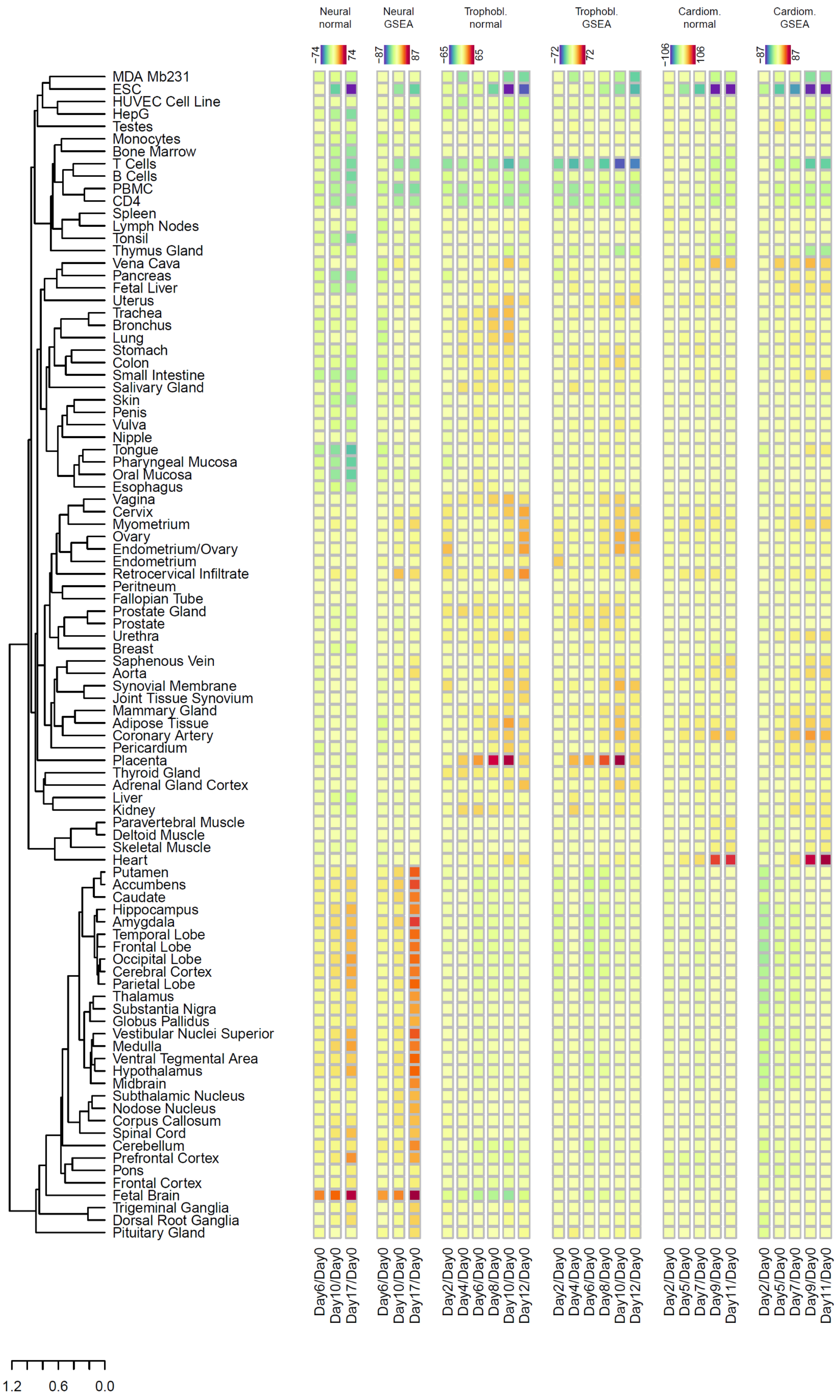

Figure S9 Heatmap comparing the differentiation time series results from the presented method to the GSEA-based implementation.

The overall results are similar in both methods as exemplified by the analysis of the three differentiation time series, but some differences can be observed. Most notably is the strongly negative T-Cell score of the trophoblast differentiation in the GSEA implementation, having an even higher absolute value than the ESC score.
